# Progress in DFT Studies of BiOF: Crystals, Defects, Doping and Heterojunctions

**DOI:** 10.3390/molecules31162935

**Published:** 2026-08-21

**Authors:** Shuili Zhang, Chao Wang, Xiong Zhang, Pengju Li

**Affiliations:** 1 School of Physics and Electronic Information, Yan’an University, Yan’an 716000, China; wangchao@stu.yau.edu.cn (C.W.); zhangxiong@yau.edu.cn (X.Z.); yadxlipengju@stu.yau.edu.cn (P.L.); 2 Shaanxi Key Laboratory of Intelligent Processing for Big Energy Data, Yan’an University, Yan’an 716000, China

**Keywords:** BiOF, DFT, defect, doping, heterojunction, photocatalysis

## Abstract

BiOF has emerged as a promising functional material for photocatalysis, electrochemical energy storage, and ion adsorption due to its unique layered structure, excellent chemical stability, and tunable electronic properties. Density functional theory (DFT) calculations provide in-depth theoretical insights into the crystal structure, intrinsic defects, doping modification, and heterostructure construction of BiOF. This review systematically summarizes the recent DFT research progress of BiOF systems. Computational results reveal the lattice characteristics, bandgap features, built-in electric field distribution and facet anisotropy of BiOF, and highlight the critical influence of Bi semicore states on structural relaxation and electronic modulation. Defect engineering of bismuth and oxygen vacancies can effectively optimize band structure, accelerate carrier separation and improve catalytic performance. Cation and anion doping introduce impurity energy levels to narrow the bandgap and broaden the visible-light response range. Various BiOF-based heterostructures with different band alignment modes are analyzed, and the interfacial built-in electric field dominates charge separation, while excessive formation energy and unfavorable charge transfer still restrict material optimization. This work provides a systematic theoretical reference for the rational design and performance improvement of high-efficiency BiOF-based materials.

## 1. Introduction

Global ecological degradation and global energy restructuring have become crucial research topics in modern materials and environmental chemistry [1,2,3]. Driven by industrial expansion, rapid urbanization and the booming new-energy industry [4,5,6], drawbacks of conventional energy consumption and environmental remediation modes are becoming increasingly obvious [7]. On one side, refractory organic and heavy metal pollutants widely exist in diverse wastewater [8,9,10,11,12,13], featuring strong persistence and bioaccumulation [14]; common physical, chemical and biological treatment approaches cannot fully mineralize contaminants [15,16], inducing secondary water pollution and aquatic ecological damage. Meanwhile, atmospheric overaccumulation of CO_2_, VOCs and sulfur/nitrogen oxides aggravates climate anomalies and endangers human health [17,18]. On the other side, global energy supply still depends heavily on fossil fuels; depleting fossil reserves widens the energy supply–demand gap, and fuel combustion releases massive pollutants to worsen ecology and form a vicious cycle [19]. Therefore, developing green, low-cost and high-efficiency remediation and energy-conversion technologies plus low-carbon pollution-control systems has become a global research hotspot [20,21,22]. Semiconductor photocatalysis and electrochemistry stand out due to their solar-driven pollutant mineralization and clean-energy preparation capacity [23,24,25]. Nevertheless, classic TiO_2_/ZnO photocatalysts suffer from a wide bandgap and poor visible-light utilization (visible light > 45% of the solar spectrum) [22], pushing the exploration of high visible-light-responsive semiconductor materials as a mainstream research direction [26].

Among semiconductors, nontoxic, structurally tunable Bi-based materials gain extensive research interest [27,28]. Layered BiOX (X = F, Cl, Br, I) bismuth oxyhalides possess alternating [Bi_2_O_2_]^2+^ and halogen stacking structures with intrinsic built-in electric field and excellent oxidation performance [29,30]. Unlike other BiOX compounds, BiOF exhibits unique physicochemical properties owing to the high electronegativity and small ionic radius of fluorine, delivering superior thermal, mechanical and anticorrosion stability [31,32,33]. Existing BiOF experiments mainly focus on morphology control, doping and heterojunction fabrication [34,35,36], yet macroscopic tests fail to clarify atomic-scale structure–activity rules, resulting in high experimental trial cost and ambiguous catalytic mechanism interpretation.

As a mainstream computational tool, DFT enables atomic-scale simulations of the crystal structure of BiOF, electronic structure and surface adsorption parameters based on quantum theory [37], quantitatively uncovering doping modulation, interfacial charge transfer and catalytic reaction thermodynamics to accelerate the development of modified BiOF photocatalysts [38,39]. A large number of scattered DFT investigations have systematically explored lattice relaxation, defect formation energy, element doping and heterojunction interface characteristics of BiOF, yet these independent studies adopt inconsistent computational parameters and lack unified comparative analysis [40]. For instance, facet-dependent calculations reveal that pH-regulated (101)-oriented BiOF nanosheets possess lower formation energy of oxygen vacancies (OVs) and superior rhodamine B degradation capacity compared with (001) crystal planes [41]. Consistent with this conclusion, engineered OVs on the BiOF (101) surface can introduce intermediate energy levels and extend carrier lifetime, thereby significantly promoting PFOA pollutant elimination [42]; both studies confirm the vital role of OV defects on the (101) facet, but they fail to discuss the influence of different exchange-correlation functionals on vacancy formation energy. In heterojunction research, the dual-oxygen-vacancy-modified S-scheme BiOBr/BiOF_x_I_1−x_ composite achieves improved CO_2_ photoreduction activity relying on the I^−^/I^0^ redox cycle [43]. In terms of cation doping, In^3+^ substitution for Bi^3+^ effectively narrows the intrinsic wide bandgap of BiOF without generating deep carrier traps, which optimizes visible light absorption performance [44]. Additionally, theoretical calculations on terminal surface configurations further summarize the stability rule of the BiOF (001) facet under different environmental conditions. Despite abundant separate theoretical outcomes, existing DFT reports lack standardized calculation protocols and systematic cross-comparison between theoretical predictions and experimental characterizations, which restricts the unified understanding of BiOF modification mechanisms.

The three aforementioned review articles, by Erbland et al. [45] on bismuth-based oxyfluorides, by Xiong et al. [46] on bismuth-rich bismuth oxyhalides, and by Sabuj et al. [47] on experimental and theoretical exploration of BiOX, approach photocatalysis from different perspectives, yet all fail to recognize the irreplaceable advantages of DFT theoretical simulation and lack a systematic collation of first-principles data. Erbland et al. [45] merely enumerate macroscopic photocatalytic performances and structural modification strategies, without employing DFT to quantitatively compare the deviations among various exchange-correlation functionals or to analyze the root causes of computational errors arising from pseudopotential choices, basis-set truncation, or van der Waals corrections, thereby missing the opportunity to establish reliable calculation protocols for this highly polar system. Xiong et al. [46] summarize a wealth of experimental tuning strategies but stop at phenomenological descriptions; they do not adopt DFT to uncover the atomistic origins of the enhanced activity, such as charge transfer at surface-active sites, the formation energetics of intrinsic defects, or the band edge alignment after doping, leaving the mechanistic links between composition and performance largely speculative. Sabuj et al. [47] does include DFT calculations, yet its scope is confined to ideal perfect bulk crystals of the three stoichiometric phases; this approach does not capture the full predictive power of DFT for defect engineering, strain effects, or heterojunction band bending, nor does it benchmark different functionals against experimental bandgaps to clarify the inherent limitations of each approach. All three reviews merely compile scattered literature records without critical cross-comparison of the reported computational data, lack comprehensive summary tables that bridge theoretical predictions with experimental observables, and have not proposed unified, standardized DFT calculation guidelines specifically tailored for BiOF or Bi-rich oxyhalide systems; there is an urgent need to ensure reproducibility and fair comparison across future theoretical studies.

Moreover, published reviews either summarize overall BiOX experimental progress or generalize DFT application across diverse Bi-based materials [48], while no specialized review systematically concludes DFT simulation for pure BiOF, missing systematic sorting on its exclusive structure, modification mechanism, and theoretical model, and hindering subsequent BiOF research innovation and application development [49,50,51]. It is critical to elaborate the unique experimental–complementary strengths of DFT; it can quantitatively capture formation energy, interfacial potential and orbital interaction parameters that cannot be directly measured by in situ characterization, decode the regulation rule of built-in electric fields in layered materials at atomic scale, and quantitatively interpret the intrinsic reasons for inconsistent experimental observations from different research teams, which constitutes the core novelty of this review.

Against this research background, this review centers on DFT investigations of pristine and modified BiOF. We summarize global theoretical advances, introduce the intrinsic structure and fundamental electrical as well as optical properties of bulk BiOF, and systematically review DFT studies on defect engineering, elemental doping, heterojunction construction and facet regulation, along with corresponding modulation rules of electronic, optical and photocatalytic performances. An independent subsection focusing on functionals, basis sets, and pseudopotentials is supplemented to compare their pros and cons for BiOF simulation, and a complete set of standardized DFT calculation workflows suitable for heavy bismuth-containing systems is proposed; summary tables integrating experimental and DFT data are attached at the end of each chapter for intuitive cross-verification. We further compare the merits and defects of various DFT parameters for BiOF simulation and interpret the microscopic catalytic pathways of BiOF in environmental governance and energy conversion. Meanwhile, we outline existing bottlenecks and controversies in current DFT research, and emerging technologies, including machine learning-assisted DFT and multi-scale and dynamic pathway simulation, are prospected for the future rational design of BiOF [52,53].

This review aims to construct a complete “structure–electronics–performance” theoretical system for BiOF to fill a relevant review gap, accompanied by supplementary calculation data on BiOF lattice, optical and thermodynamic parameters. This review is divided into six core sections: crystal structure, intrinsic defects, elemental doping, heterojunction properties, catalytic mechanism, conclusion, and outlook. It supplies systematic theoretical guidance for BiOF experimental optimization and composite construction, facilitating its industrialization in pollution remediation and clean energy storage/conversion.

## 2. The Structure and Basic Performance of Intrinsic BiOF

BiOF is a direct bandgap semiconductor. Its layered crystal structure consists of alternately stacked [Bi_2_O_2_]^2+^ layers and double F^−^ layers. The inhomogeneous interlayer charge distribution generates a built-in electric field along the *c*-axis, which facilitates the separation of photogenerated charge carriers. The optical absorption edge of BiOF lies in the ultraviolet region, and the material is nearly transparent under visible light, exhibiting strongly anisotropic optical responses. Mechanically, BiOF presents a counterintuitive combination of high rigidity and low hardness, which originates from the strong ionic character of Bi-F bonds. Crystal facet engineering enables the preferential exposure of highly active (002) and (101) facets. These facets are rich in OVs and thus deliver remarkably enhanced photocatalytic performance, while surface holes act as the dominant active species for dye degradation. The above intrinsic properties have been systematically elucidated at the atomic scale via first-principles calculations.

### 2.1. The Crystal Structure of BiOF

**Figure 1:**
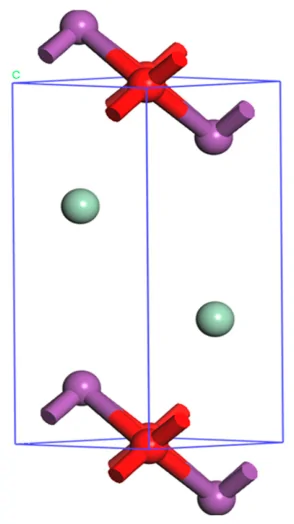
The tetragonal crystal structure of BiOF.

The atomic arrangement of crystals serves as the fundamental basis for understanding their macroscopic physical properties. BiOF crystallizes in the tetragonal crystal system with the space group *P4/nmm* and adopts a typical layered PbFCl-type structure [54,55]. Figure 1 illustrates the tetragonal layered crystal structure of BiOF for intuitive observation of structural features. It can be clearly observed that the crystal is constructed by alternating stacking of [Bi_2_O_2_]^2+^ layers and double F^−^ layers along the *c*-axis. The stacking sequence follows the pattern of -F-Bi-O-Bi-F-. Weak van der Waals (vdW) forces dominate the interactions between adjacent layers [56,57]. Compared with BiOCl, BiOBr, and BiOI, BiOF possesses the smallest interlayer distance. This phenomenon is consistent with the fact that F^−^ has the smallest ionic radius. Meanwhile, this structural characteristic indicates that BiOF exhibits the highest interlayer rigidity and anti-exfoliation capability.

After clarifying the basic crystal configuration, lattice parameters were further quantitatively characterized. The ionic radius of F^−^ is approximately 1.33 Å, which is the smallest among all halogen ions. Accordingly, BiOF shows evidently smaller lattice constants than other BiOX compounds. The lattice parameters optimized with the GGA PBE functional are a = b = 3.747 Å and c = 6.226 Å. These values are in excellent agreement with experimental results of a = 3.760 Å and c = 6.230 Å, with a relative error lower than 1% [33,58]. This difference directly reflects the lattice expansion effect caused by the increase in halogen ionic radius as we move from BiOF to other BiOX materials [32]. Notably, BiOF has a c/a ratio of around 1.66, which ranks the highest within the BiOX family. This result demonstrates its most distinct layered structure and the weakest interlayer electron cloud overlap. Such characteristics exert vital influences on electronic band structures and optical anisotropy. Apart from lattice constants, bonding characteristics are also essential to reveal the correlation between structure and performance. The Bi-O bond length in BiOF is about 2.31 Å with a strong covalent nature. The Bi-F bond length ranges from 2.75 Å to 2.79 Å and presents a dominant ionic character [54,55]. In terms of spatial orientation, Bi-O bonds form distorted tetragonal pyramidal coordination inside the [Bi_2_O_2_]^2+^ layers. Bi-F bonds extend perpendicular to the layer plane and connect adjacent fluorine layers. The anisotropic bonding features provide a solid structural foundation for the subsequent analysis of directional dependence in the electrical, optical, and mechanical properties of BiOF.

The alternating arrangement of positively charged [Bi_2_O_2_]^2+^ slabs and electronegative double F^−^ layers generates spontaneous charge polarization along the *c*-axis of the BiOF lattice, forming a stable intrinsic internal electric field (IEF) perpendicular to the laminar plane. This IEF serves as a core structural feature of BiOF and will be referenced in subsequent sections when discussing carrier separation and photocatalytic performance.

### 2.2. Electrical Properties of BiOF

**Figure 2:**
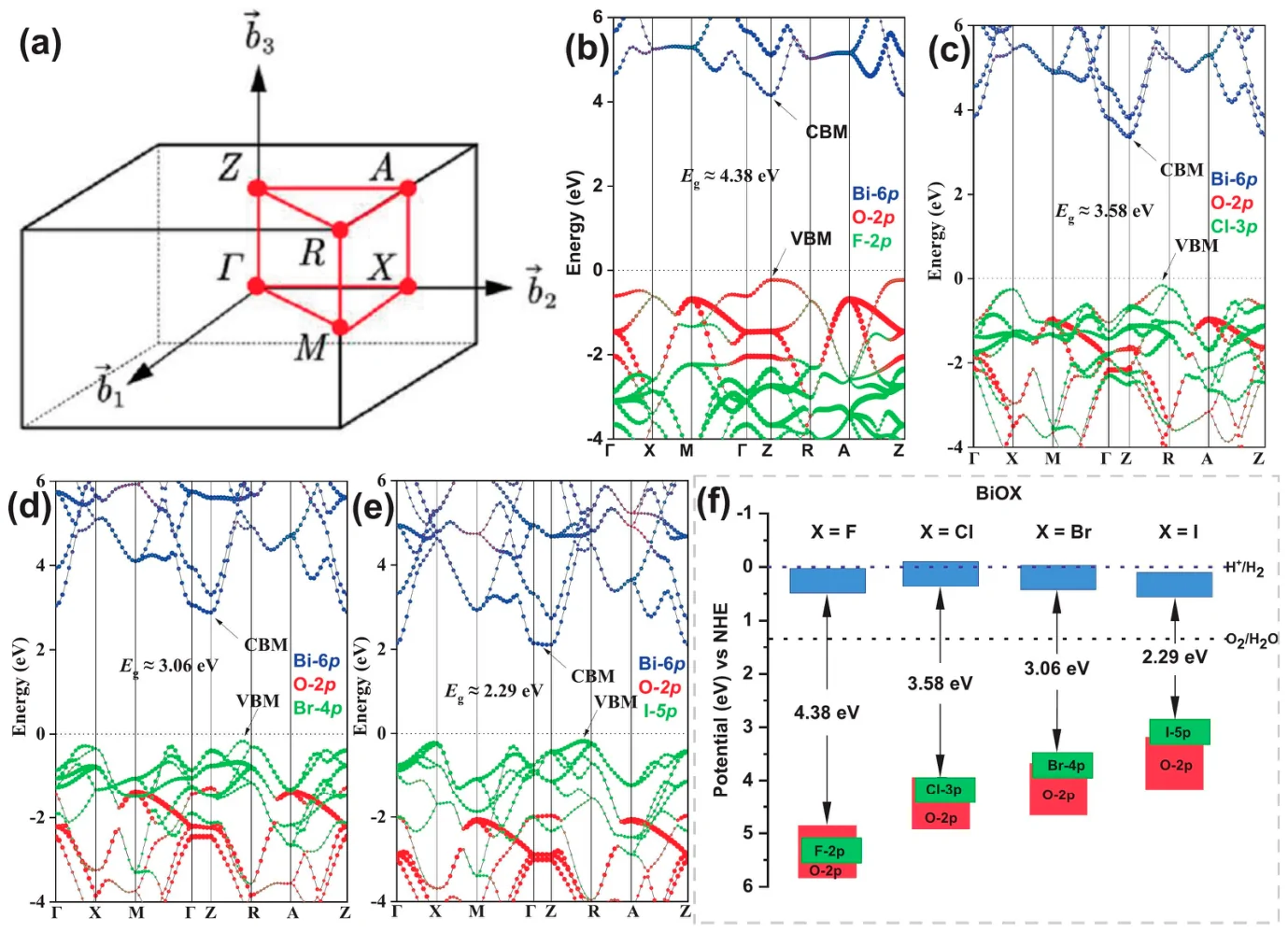
(**a**) The Brillouin zone of BiOX. (**b**–**e**) The band structures of BiOX. (**f**) Schematic valence and conduction band structures of BiOX. Reprinted from Ref. [56] with permission from Elsevier.

After determining the crystal structure, the electrical properties of BiOF are analyzed in this section from three aspects, including band structure characteristics, density of states (DOS) composition, and charge distribution. Figure 2a–e presents the band structure, projected density of states (PDOS), and differential charge density of BiOF, which visually support the following analysis of bandgap type, orbital contributions, and bonding characteristics.

In terms of bandgap type, most theoretical calculations confirm that BiOF is a direct bandgap semiconductor. Both the valence band maximum (VBM) and conduction band minimum (CBM) are located at or near the Z point [54,55]. Several studies also classify BiOF as a nearly direct bandgap material [33]. Notably, the calculated bandgap exhibits strong functional dependence, which is a typical limitation of semi-local and hybrid DFT methods for wide-gap bismuth halide semiconductors: the GGA-PBE functional ranges from 3.13 eV to 3.29 eV. This wide scatter (spanning ~1.7 eV) highlights that the choice of functional and whether calculations use relaxed or experimental lattice parameters can shift the gap by over 1.5 eV for the same material. The values obtained using the HSE06 hybrid functional are between 4.18 eV and 4.47 eV. The calculation based on the mBJ potential gives a bandgap of 4.85 eV [32,33,59]. The experimentally measured optical bandgap falls in the range of 3.64 eV to 3.93 eV [32], lying in the middle of the three sets of theoretical values, which reveals the inherent band deviation error of mainstream functionals for BiOF systems. This counterintuitive agreement arises from error cancelation; PBE underestimates the gap while lattice expansion upon relaxation further reduces it, bringing PBE accidentally closer to experiment; HSE06 and GW, though theoretically more rigorous, lack this error cancellation and deviate more from the measured value.

For comparison, the experimental bandgaps of BiOCl, BiOBr, and BiOI are approximately 3.4 eV, 2.8 eV, and 1.9 eV, respectively (Figure 2f) [32,58]. The overall bandgap decreases monotonically by about 2.3 eV from BiOF to BiOI. This trend originates from the gradual decline in halogen electronegativity. The energy of halogen n*p* orbitals increases accordingly. Orbital hybridization between halogen n*p* and O 2*p* shifts the VBM upward. The CBM is primarily contributed by Bi 6*p* orbitals and shows negligible variation. The combined effects lead to the continuous narrowing of the bandgap [32,56].

The band composition and DOS distribution are discussed subsequently. DOS analysis reveals [54,55,60] that the VBM of BiOF mainly originates from the hybridization of O 2*p* and F 2*p* orbitals, and O 2*p* orbitals play the dominant role. The CBM is almost entirely composed of Bi 6*p* orbitals [54,61]. Inclusion of spin–orbit coupling lowers the Bi 6*p*-derived CBM by approximately 0.3–0.4 eV, resulting in a net bandgap reduction of ~0.39 eV for BiOF, consistent with hybrid DFT + SOC calculations. The specific orbital composition enables the spatial separation of photogenerated charge carriers. After photoexcitation, electrons are mainly localized in the [Bi_2_O_2_] layers, while holes are concentrated in fluorine layers [32,56]. This intrinsic orbital partition characteristic matches the layer-based charge polarization described in Section 2.1 perfectly, which, combined with the built-in electric field along the *c*-axis induced by the layered structure of BiOF, this characteristic effectively accelerates the spatial separation of electron–hole pairs and suppresses their recombination. The anisotropic bonding environment, strong covalent bonding within the [Bi_2_O_2_]^2+^ slab and weak van der Waals interaction between adjacent layers, as illustrated in Section 2.1, constitutes the fundamental origin of electrical anisotropy in BiOF. This anisotropy is quantitatively reflected in carrier effective masses: the electron effective mass is 0.5 m_e_ along the out-of-plane direction (Z→Γ) versus 1.0 m_e_ in the in-plane direction (Z→R), directly correlating with the weaker interlayer coupling in the layered PbFCl-type structure.

Finally, the effective charge of Bi gradually decreases and the absolute value of negative charge on halogen atoms declines when halogen elements change from F to I. This phenomenon indicates a continuous increase in the covalent component of Bi-X bonds [61]. The changing trend is consistent with the chemical origin of bandgap evolution.

### 2.3. Optical Properties of BiOF

**Figure 3:**
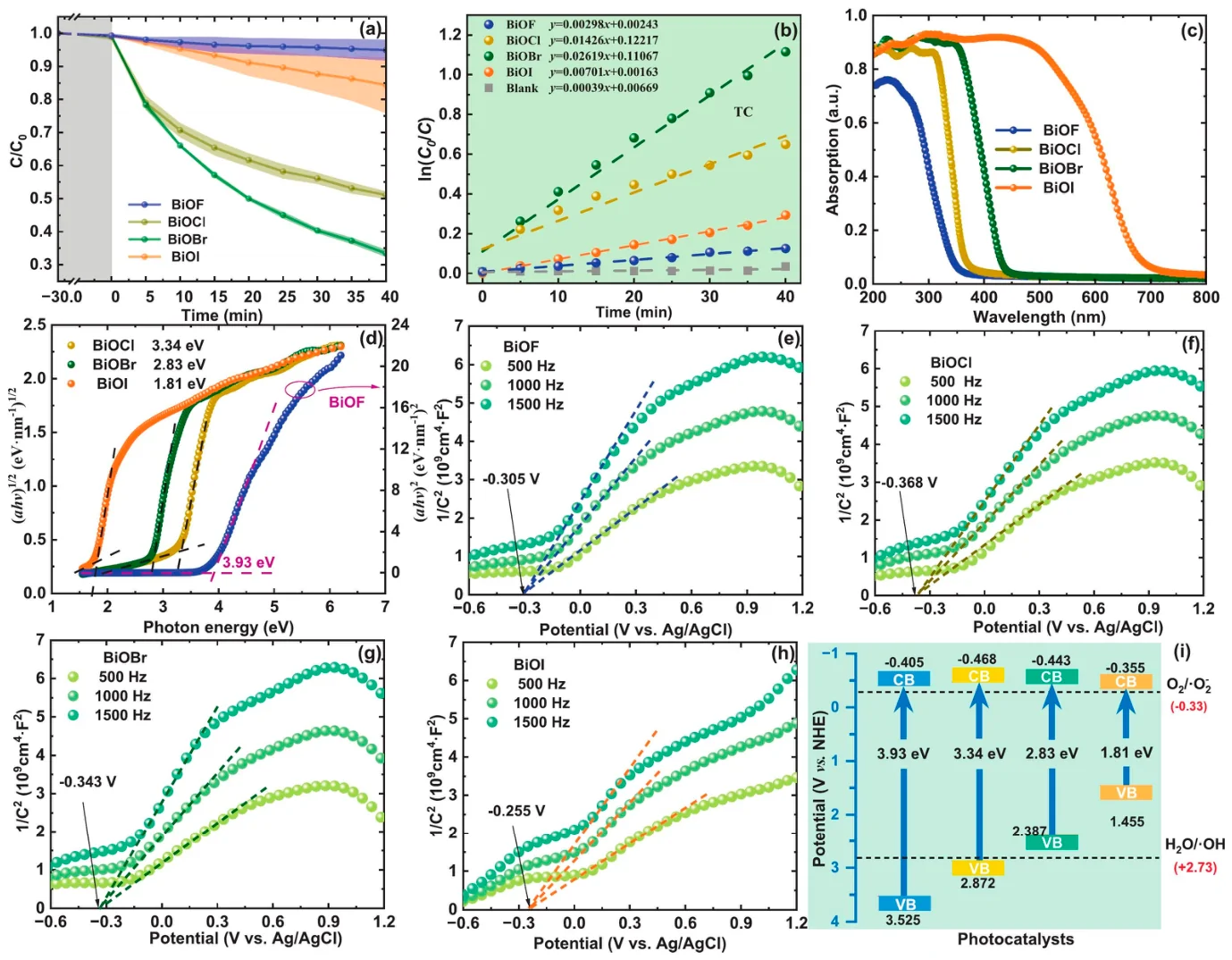
Photocatalytic activities of (**a**) BiOX and (**b**) its photocatalytic kinetics. (**c**) UV–vis DRS profiles of BiOX and (**d**) its Tauc curves for Eg. (**e**–**h**) Mott–Schottky plots. (**i**) The illustrated band scheme. Reprinted from Ref. [56] with permission from Elsevier.

Electrical properties determine the optical response of materials. Therefore, the optical behaviors of BiOF are further explored after the analysis of electronic band structures. Figure 3 presents the UV–vis diffuse reflectance spectra (DRS) of BiOF, BiOCl, BiOBr, and BiOI, showing the absorption edges and optical bandgaps of the four bismuth oxyhalides for direct comparison. As discussed in Section 2.2, the closer agreement of the GGA-PBE value with experiment arises from error cancelation rather than physical accuracy, whereas the BSE level provides a more physically meaningful description by explicitly including electron–hole interactions. The optical absorption spectra calculated via the Bethe–Salpeter equation method take excitonic effects into consideration. The first absorption peak is located at approximately 3.75 eV, which is closer to experimental results than the data obtained from the independent particle approximation [59]. Due to the wide bandgap, BiOF hardly absorbs visible light with wavelengths longer than around 400 nm and thus shows optical transparency [58]. The absorption edge of BiOF is blueshifted relative to BiOCl, BiOBr, and BiOI, consistent with the trend of bandgap narrowing with increasing halogen atomic number discussed in Section 2.2. In summary, the optical absorption of BiOF is restricted to the ultraviolet region, and its absorption edge matches well with the calculated bandgaps. The BSE calculation further verifies the essential role of excitonic effects in the light absorption process. BiOF also exhibits obvious directional dependence in optical properties. Both the absorption coefficient and the real part of the dielectric function along the (100) direction within the layers are higher than those along the (001) direction between layers. For instance, the static value of dielectric function ε_1_(0) is about 4.82 along the (100) direction and 4.34 along the (001) direction [33]. This optical anisotropy directly reflects the structural anisotropy described in Section 2.1: strong covalent Bi–O bonds within the [Bi_2_O_2_]^2+^ slabs enhance in-plane polarizability, while weak van der Waals interactions between adjacent layers reduce out-of-plane response. Such anisotropy is also manifested in the direction-dependent effective masses, with values of 0.5 m_e_ along the out-of-plane direction (Z→Γ) and 1.0 m_e_ along the in-plane direction (Z→R), as well as in the anisotropic absorption coefficient, which exhibits higher values for in-plane polarization in the low-energy region. The reflection spectra show that BiOF maintains a reflectance below 10 percent in the low-energy region, which corresponds to its transparent feature. A series of reflection peaks appear in the high-energy region, ranging from 10 eV to 20 eV [61].

A critical comparison between theoretical predictions and experimental optical data reveals several key discrepancies. The static dielectric constant varies significantly with method: PBE gives ~4.82 (xx) and ~4.34 (zz) [33], whereas BSE yields a much lower isotropic value of ~3.80; comparison with BiOCl suggests the BSE value may be more physically reliable, as it accounts for electron–hole interactions that reduce screening [59]. The optical absorption edge from BSE (~3.75 eV) agrees well with the experimental onset, while GGA-PBE places the edge too low [33] and HSE06 places it too high [59], mirroring the bandgap behavior discussed in Section 2.2. Independent-particle calculations (GGA/HSE without BSE) fail to reproduce the low-energy peak positions, whereas BSE captures the first absorption peak at 3.75 eV [59]. The computed optical anisotropy, with higher response along the in-plane direction [33,61], has not been directly validated by polarized single-crystal measurements, representing a gap between theory and experiment. Overall, the most reliable theoretical description requires hybrid functionals + SOC + BSE; GGA-PBE alone, despite its accidental bandgap agreement, fails to capture the optical spectra quantitatively.

Overall, BiOF shows nearly no absorption in the visible light region, owing to its having the widest bandgap among bismuth oxyhalides. This limitation makes pristine BiOF unsuitable for standalone visible-light photocatalysis under solar irradiation. Meanwhile, it brings unique advantages for deep ultraviolet optoelectronic devices, high-transmittance ultraviolet optical windows, and photocatalytic reactions requiring high energy photons, such as the degradation of perfluorinated compounds. This UV-only absorption characteristic also explains why most BiOF modification strategies, including doping, defect engineering, and heterojunction construction, aim to extend its light-response range into the visible region.

Table 1 summarizes the structural, electronic, and optical parameters of BiOF from various DFT calculations and experimental measurements. The data cover crystal structure, bandgaps from different functionals, bond lengths, atomic charges, dielectric properties, and carrier effective masses, illustrating the strong dependence of the calculated bandgap on the chosen theoretical method and the optical anisotropy of this layered material.

### 2.4. The Crystal Facets and Adsorption Properties of BiOF

Previous sections have discussed the bulk properties of BiOF, including crystal structure, electronic band, optical response, mechanical performance, and thermodynamic characteristics. For photocatalytic materials, however, catalytic reactions primarily take place on material surfaces. This section combines first-principles calculations and experimental characterization to systematically investigate the facet and adsorption properties of BiOF from three perspectives: facet stability, selective exposure, and the synergistic effect of the IEF with surface adsorption characteristics.

The layered structure of BiOF makes the (001) facet, which is perpendicular to the *c*-axis, the most easily exposed surface and gives it superior thermodynamic stability. Four types of (001) surface models were constructed in theoretical calculations, including single halogen termination, double halogen termination, oxygen termination, and bismuth termination. Surface energy calculations reveal that surfaces with single halogen termination possess the lowest surface energy across all BiOX systems and thus present the highest stability. The overall stability decreases in the order of BiOI, BiOF, BiOBr, and BiOCl. For BiOF, specifically, the low surface energy of the single fluorine-terminated (001) surface originates from its stable electronic structure. The absence of dangling bonds on the outermost atomic layer also prevents the generation of additional surface dipole moments [57]. Notably, the double-fluorine-terminated (001) surface of BiOF also exhibits considerable stability [57]. This result indicates that vdW interactions within double fluorine layers can effectively stabilize the surface structure. A critical point worth emphasizing is that the surface energy values obtained from DFT depend sensitively on the choice of exchange-correlation functional and the inclusion of van der Waals corrections; Cabeza et al. [57] employed the DFT-D3 method, which is essential for accurately describing the interlayer interactions in BiOF. Without such corrections, the relative stability order of different terminations would be significantly altered.

**Figure 4:**
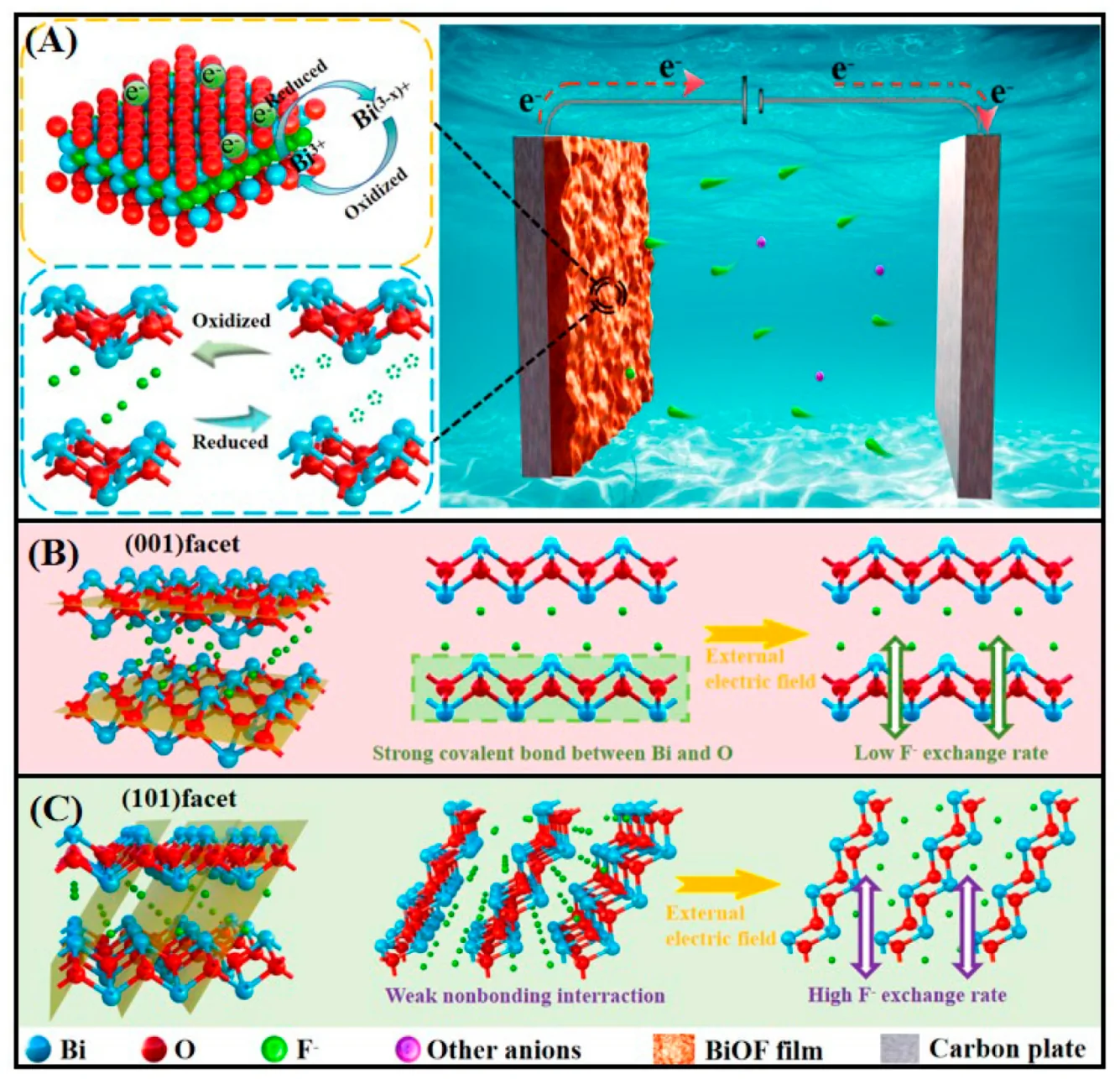
Schematic diagram depicting the adsorption and desorption processes of F- (**A**), (001)facet (**B**), and (101)facet (**C**). Reprinted from Ref. [62] with permission from Elsevier.

On this basis, experimental studies further explore the exposure of highly active facets and their influence on photocatalytic performance. Regular BiOF nanosheets were successfully synthesized by adjusting hydrothermal reaction conditions at a pH value of 9, a temperature of 160 degrees Celsius, and a reaction duration of 12 h. Scanning electron microscopy images show that the obtained products present a tetragonal thin-sheet morphology with a side length of approximately 4 μm and a thickness of around 0.6 μm. High-resolution transmission electron microscopy and selected area electron diffraction results further confirm that these nanosheets preferentially grow along the (110) and (100) directions, which leads to large-scale exposure of the (002) facet. Geometric calculation demonstrates that the exposure ratio of the (002) facet reaches up to 75.4 percent [58]. Photocatalytic performance evaluations show that the as-prepared BiOF nanosheets exhibit excellent degradation activity toward RhB under ultraviolet irradiation. The degradation efficiency reaches 79.3 percent within 60 min, and the apparent rate constant is 0.02534 min^−1^, which is 3.88 times that of commercial rutile titanium dioxide [58]. It is worth mentioning that the specific surface area of BiOF is only 2.1 m^2^ g^−1^, which accounts for merely 4.2 percent of rutile titanium dioxide. Despite the much smaller specific surface area, BiOF delivers higher photocatalytic activity. This counterintuitive phenomenon proves the dominant role of exposed highly active facets over mere surface area effects. However, a direct quantitative comparison between the (002)-exposed nanosheets [58] and the (101)-exposed nanoplates [41] reveals that the latter exhibit even higher photocatalytic rates (0.098 min^−1^ vs. 0.025 min^−1^), suggesting that the (101) facet is intrinsically more reactive than the (002) facet for RhB degradation. This difference arises from the higher density of surface oxygen vacancies on the (101) facet, as confirmed by EPR measurements [41]. Guo et al. [62] further confirmed that high (101) facet exposure enhances F^−^ capture: DFT calculations give an adsorption energy of −3.91 eV on (101) vs. −3.16 eV on (001). The BOF-C sample delivers an adsorption capacity of 24.14 mg·g^−1^ (0.9 V) with high selectivity (Figure 4A–C). Notably, the adsorption energy difference of 0.75 eV between the two facets directly correlates with the experimentally observed selectivity, demonstrating that DFT calculations can quantitatively predict facet-dependent ion-capture performance.

Apart from facet stability and selective exposure, surface adsorption characteristics also govern the pathway and efficiency of photocatalytic reactions. Radical trapping experiments show that the degradation efficiency drops from 79.3 percent to approximately 30 percent after adding hole scavengers and decreases to about 65 percent with the introduction of superoxide radical (·O_2_^−^) scavengers [58]. The results demonstrate that photogenerated holes (h^+^) act as the main active species for RhB degradation over BiOF, while ·O_2_^−^ serves as a secondary active species. This feature is closely related to the high electronegativity of fluorine atoms. Surface fluorine atoms tend to capture holes and make holes the major oxidizing agent, which changes the conventional reaction pathway dominated by ·O_2_^−^ in general n-type semiconductors. A critical analysis of the literature reveals the following apparent contradiction: for (101)-facet-dominated BiOF nanoplates, both h^+^ and ·O_2_^−^ contribute significantly [41], whereas for (002)-facet-dominated nanosheets, h^+^ is the sole dominant species [58]. This discrepancy suggests that the exposed facet not only determines the surface adsorption energy but also dictates the reactive species generation pathway, which is a crucial consideration for designing facet-specific photocatalysts. In addition, theoretical calculations indicate that the BiOF (001) surface presents moderate adsorption energy for small molecules such as water and oxygen. Such adsorption strength facilitates the activation of reactant molecules and avoids surface poisoning caused by excessive adsorption. This property endows BiOF with great potential for application in photocatalytic water treatment.

As described in Section 2.1, the IEF along the c-axis drives photogenerated electrons toward the [Bi_2_O_2_]^2+^ layers and holes toward the fluorine layers on the exposed surfaces, achieving spatial charge separation and greatly reducing carrier recombination [58]. Electrochemical impedance spectroscopy measurements confirm this mechanism: the Nyquist plot of BiOF shows a much smaller semicircle radius compared with rutile TiO_2_, indicating the lower interfacial charge transfer resistance and higher carrier separation efficiency of BiOF. However, it should be noted that the IEF strength is highly dependent on the surface termination and the presence of defects; fluorine vacancies, for instance, can partially destroy the IEF and thereby reduce the charge separation efficiency [63]. This sensitivity implies that experimental synthesis conditions must be carefully controlled to preserve the IEF.

Table 2 compares the key properties of different BiOF facets, including surface energy, adsorption energy, exposure ratio, dominant reactive species, and photocatalytic rate. The (001) facet is the most thermodynamically stable but shows limited photocatalytic activity. In contrast, the (101) facet exhibits stronger F^−^ adsorption and a significantly higher photocatalytic rate (0.098 min^−1^) due to the synergistic effect of h^+^ and ·O_2_^−^ species, while the (002) facet with 75.4% exposure ratio shows moderate performance (0.02534 min^−1^). These results highlight the critical role of facet engineering in optimizing the photocatalytic efficiency of BiOF.

### 2.5. Basis Set and Pseudopotential

In plane-wave pseudopotential (PW-PP) calculations, the choice of pseudopotential type critically influences the accuracy of BiOF electronic structures. Two categories of pseudopotentials have been extensively employed in BiOX studies: ultrasoft pseudopotentials (USPP) and norm-conserving pseudopotentials (NCPP), as well as the projector-augmented wave (PAW) method in recent years [54,55]. Huang and Zhu systematically compared USPP and NCPP for BiOX systems. In their USPP scheme, Bi was described with only 6*s* and 6*p* states (five valence electrons), whereas the NCPP additionally included the semicore 5*d* states (15 electrons in total). Their results demonstrate that the inclusion of Bi 5*d* states significantly expands the bandgap of BiOF from 3.12 eV to 3.22 eV, and similar expansions of 0.21, 0.23, and 0.22 eV were observed for BiOCl, BiOBr, and BiOI, respectively [54]. This underscores that the proper treatment of Bi 5*d* semicore states is essential for achieving reliable electronic structures in BiOF calculations. Excluding Bi 5*d* semicore states underestimates the BiOF bandgap by ~0.1 eV, which cannot be ignored for quantitative band alignment analysis.

The PAW method, which combines the accuracy of all-electron methods with the efficiency of pseudopotential approaches, has become increasingly adopted for BiOF studies. Ganose et al. employed PAW pseudopotentials with the HSE06 hybrid functional and explicitly included spin–orbit coupling (SOC), using a plane-wave cutoff of 520 eV and a k-point density of 0.04 Å^−1^ [32]. Their calculations reveal that SOC reduces the bandgap of BiOF by 0.39 eV, with reductions of 0.24, 0.24, and 0.22 eV for BiOCl, BiOBr, and BiOI, respectively [32]. Barhoumi and Said further employed PAW within the GW approximation, utilizing a 500 eV cutoff and a 6 × 6 × 4 k-point mesh [59]. They noted that DFT methods, particularly LDA and GGA, significantly underestimate the BiOX bandgaps, necessitating higher-level approximations such as GW or hybrid functionals for accurate predictions.

Beyond the treatment of Bi 5*d* states, the role of Bi 5*f* states deserves attention. Huang and Zhu investigated the influence of Bi 5*f* states on BiOX electronic structures using norm-conserving pseudopotentials that incorporated the 5*d*, 5*f*, 6*s*, and 6*p* states of Bi (15 electrons total) [55]. Although the Bi 5*f* states are primarily located above the conduction bands and contribute negligible density within the displayed energy range, their inclusion substantially affects atomic relaxation behavior. Specifically, relaxation with Bi 5*f* states yielded opposite atomic displacement directions and expanded bandgaps compared to results without Bi 5*f* states [55]. For unrelaxed BiOF, a direct bandgap of 3.25 eV was obtained, whereas relaxation expanded it to 3.41 eV. Löwdin population analysis further indicates small but non-negligible occupancies of Bi 5*f* states (0.27–0.33 eV), confirming that their inclusion improves computational accuracy.

The layered structure of BiOX, composed of [Bi_2_O_2_]^2+^ layers and double halogen layers stacked along the c-axis via weak van der Waals (vdW) interactions, necessitates the inclusion of vdW corrections for the accurate description of interlayer interactions. Ganose et al. emphasized that the nonlocal dispersion correction (DFT-D3) is vital for achieving accurate equilibrium geometry, noting that without vdW interactions, the error in the c parameter can grow to as much as 1 Å [32]. Cabeza et al. also confirmed the necessity of DFT-D3 in surface energy and cleavage energy calculations for BiOX (001) surfaces. Barhoumi and Said employed the DFT-D2 method of Grimme to treat vdW interactions, finding that although vdW corrections have a relatively minor effect on bandgaps (approximately 0.01–0.03 eV), they are essential for optimizing lattice constants, particularly the c-axis parameter [59]. Dutta et al. further investigated the impact of biaxial strain on BiOX structural stability within the DFT-D3 framework, demonstrating that reliable interlayer distances cannot be obtained without vdW corrections [64].

For high-accuracy all-electron calculations, the full-potential linear augmented plane-wave (FP-LAPW) method implemented in the WIEN2k code has been applied to BiOF. Boran and Kara systematically examined various exchange-correlation functionals for BiOF using the LAPW approach [33]. In LAPW calculations, the basis set consists of radial functions within atomic spheres and plane waves in the interstitial region. For systems containing heavy Bi atoms, particular attention must be paid to the completeness of the radial basis set, particularly for atoms with p semicore states, which can be improved by adding relativistic local orbitals (RLOs). Muffin-tin radii should be carefully chosen; typical values reported in the literature include 2.34 a.u. for Bi, 1.90 a.u. for O, and 2.32 a.u. for F [64]. The plane-wave cutoff parameter R_MT_ × K_max_ is typically set to 7–8, with partial wave expansion up to l_max_ = 10–12 [64]. Boran and Kara employed an 11 × 11 × 7 Monkhorst–Pack k-point grid for self-consistent calculations, achieving well-converged results [33].

Based on the above analysis, several practical recommendations can be made for DFT calculations of BiOF systems. The PAW method is recommended as the preferred pseudopotential approach, offering a favorable balance between accuracy and computational efficiency. Bi 5*d* states must be treated as valence states, as their inclusion yields bandgap corrections of 0.1–0.3 eV. The inclusion of Bi 5*f* states, despite higher computational cost, is advisable for accurate atomic relaxation studies. Given the layered nature of BiOF, DFT-D2 or DFT-D3 vdW corrections are indispensable for obtaining reliable interlayer distances and surface energies. SOC effects must be explicitly included, as they reduce the bandgap by approximately 0.2–0.4 eV. Finally, for bandgap predictions, HSE06 or GW approximations are strongly recommended over semi-local functionals to overcome the well-known DFT underestimation of bandgaps. Table 3 summarizes the key pseudopotential and basis set combinations employed in DFT calculations for BiOF and related BiOX systems, along with their corresponding treatments of Bi semicore states, van der Waals corrections, spin–orbit coupling, and the resulting bandgaps. This comparison facilitates the selection of appropriate computational protocols for future theoretical investigations of BiOF-based materials.

In summary, the choice of pseudopotential and basis set can affect the electronic structure of BiOF as much as, or even more than, the choice of exchange-correlation functional, especially for heavy Bi atoms with active semicore and relativistic orbitals. Neglecting Bi 5*d*/5*f* states, spin–orbit coupling, or van der Waals corrections will produce misleading bandgaps, lattice constants, and interfacial charge distribution results. The PAW-D3-SOC + HSE06 combination delivers the best balance of accuracy and computational cost for BiOF theoretical simulations.

## 3. The Regulatory Mechanism of Intrinsic Defects on the Electronic Structure and Property of BiOF

Defects exert significant modulation effects on the electronic structure, photocatalytic performance, and electrochemical properties of BiOF [65]. Different from elemental doping, defect engineering tunes band structures and surface activity merely by adjusting the concentration of intrinsic point defects without introducing foreign atoms. This section discusses the regulation effects of various intrinsic defects on the electronic structure and comprehensive properties of BiOF.

Bi vacancies exhibit great potential for electrochemical energy storage. Using first-principles calculations, Yang et al. studied the structural relaxation, formation energy, electronic structure, and electrochemical properties of Bi vacancies in BiOF [63]. The results indicate that charged Bi vacancies are thermodynamically stable under certain chemical potentials and Fermi levels, whereas neutral ones are hard to form. Under O-rich or Bi-rich conditions with the Fermi level near the CBM, trivalent negatively charged Bi vacancies have the lowest formation energy and form preferentially. These defects narrow the bandgap of BiOF from 3.10 eV to 1.56 eV. Density of states analysis shows that defect states appear near the VBM, primarily originating from Bi 6*p* orbitals, and theoretical predictions suggest improved average voltage and capacity for Li-ion battery cathodes. However, the GGA-PBE calculations did not include SOC, which is known to lower Bi 6*p*-derived states by ~0.3–0.4 eV in BiOF; the predicted 1.56 eV bandgap may therefore be significantly underestimated. Moreover, the deep in-gap Bi 6*p* states could potentially act as carrier recombination centers rather than mere conductivity enhancers, an issue that requires verification with hybrid functionals and carrier lifetime assessments. Photocatalytic applications of Bi vacancies remain largely unexplored, but future studies should clarify whether these bulk defects facilitate or impede surface charge transfer before pursuing their use in photoelectrochemical or composite systems.

Research on F vacancies in BiOF is also relatively scarce. According to the calculations conducted by Yang and colleagues [63], positively charged F vacancies can remain stable under certain conditions, although their formation energy is substantially higher than that of OVs and Bi vacancies. After the introduction of F vacancies with positive one charge, the bandgap of BiOF decreases slightly from 3.10 eV to 2.92 eV, with defect states emerging near the CBM and negligible impact on the VBM structure. As defined in Section 2.1, BiOF possesses a c-axis IEF. The claim that fluorine vacancies disrupt this IEF is only a qualitative deduction without quantitative electrostatic potential verification from DFT simulations. Moreover, unlike OVs, F vacancies have not yet been experimentally detected, making their actual existence and impact largely speculative. While their high formation energy and potential disruption of charge separation render F vacancy engineering unfavorable for photocatalysis, their possible utility in fluoride-ion conduction or electrochemical ion-exchange remains unexplored. Future DFT work should employ hybrid functionals with SOC to accurately determine the defect level positions and include electrostatic potential analysis to validate the proposed modulation of the internal electric field.

OVs are the most widely studied intrinsic defects in BiOF and present the most prominent modulation effects on material performance. In terms of electronic structure, DFT calculations conducted by Li and coworkers reveal that OVs possess a favorable thermodynamic formation condition with a low formation energy of −4.18 eV. The pristine BiOF exhibits a bandgap of 3.12 eV, while the introduction of OVs reduces the bandgap to 2.65 eV. Meanwhile, a new defect state dominated by Bi 6*p* orbitals emerges within the forbidden band [66]. The introduced defect level acts as a trapping site for photogenerated electrons, which accelerates the separation of electron–hole pairs and leads to a red shift in the optical absorption edge. However, the calculated formation energy of −4.18 eV appears anomalously low, which implies spontaneous OV formation and contradicts experimental XPS/EPR observations showing only finite OVs concentrations. This discrepancy may arise from the use of a small 2 × 2 × 1 supercell without convergence testing and the omission of SOC corrections for Bi 6*p*-derived states.

**Figure 5:**
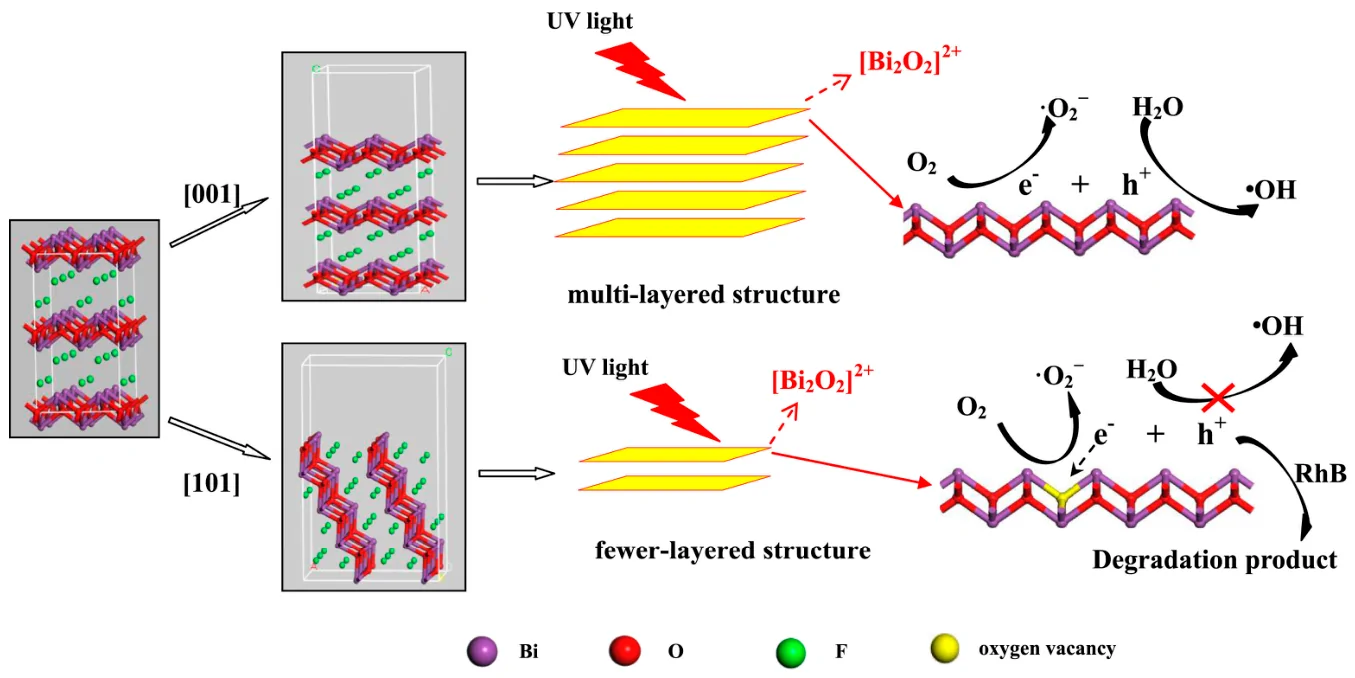
The schematic diagram of the multi-layer and single-layer structures shows the reaction mechanism. Reprinted from Ref. [41], with permission from Elsevier.

Furthermore, the concentration of surface OVs in BiOF is strongly dependent on its exposed crystal facets and can be precisely regulated by adjusting hydrothermal reaction conditions. Zhang and coworkers successfully fabricated BiOF nanoplates with tunable dominant crystal facets via modulating the pH value of the hydrothermal solution [41]. Figure 5 compares the photocatalytic mechanisms of (001)- and (101)-faceted BiOF. The (001) surface, with fewer OVs, relies mainly on ·OH and ·O_2_^−^, while the (101) surface, enriched with OVs, promotes ·O_2_^−^ generation via trapped electrons and enhances hole-mediated oxidation, resulting in superior degradation performance. Electron paramagnetic resonance (EPR) signal intensity is positively correlated with OV content, and PL as well as photocurrent characterizations further verify that OVs effectively suppress photogenerated carrier recombination. The (101)-oriented thin nanosheets, rich in surface OVs, achieve a RhB degradation rate constant of 0.098 min^−1^, which is 7.9 times that of thick (001)-oriented plates. Active species-trapping experiments reveal that ·OH and ·O_2_^−^ dominate degradation over thick plates, whereas holes and ·O_2_^−^ are the primary species for thin nanosheets [41]. This shift originates from OV-trapped electrons preferentially reducing O_2_ to ·O_2_^−^ while suppressing ·OH production, thereby favoring hole-mediated oxidation. Notably, a direct DFT comparison of OV formation energies on the (101) vs. (001) facets, which would clarify why the (101) surface preferentially hosts more vacancies, has not yet been reported. Additionally, the precise energy position of the Bi 6*p*-derived defect level was not determined in the original study; this distinction is critical because deep traps typically act as recombination centers rather than separation promoters. Future calculations using HSE06 + SOC with systematic supercell-size convergence are needed to resolve these uncertainties.

**Figure 6:**
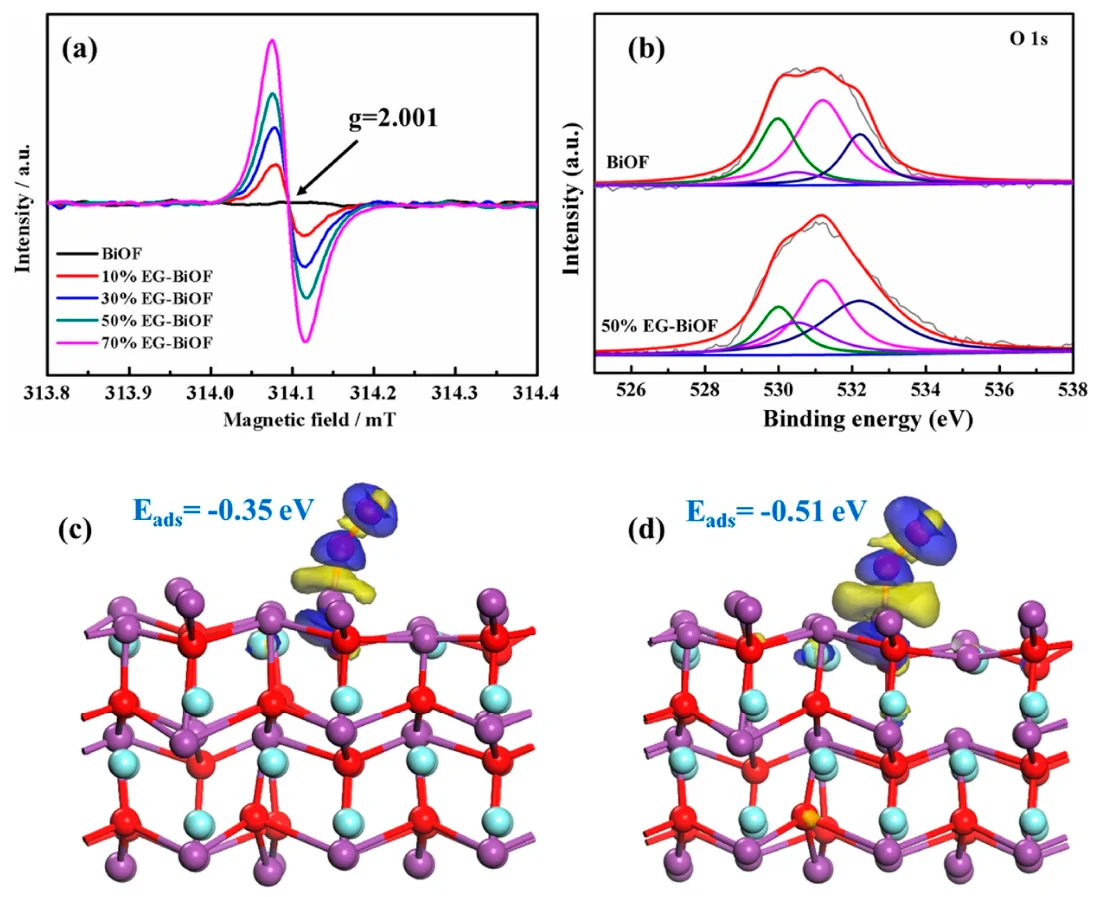
(**a**) EPR spectra of the products prepared with different amounts of EG; (**b**) O 1*s* XPS spectra of BiOF and 50% EG-BiOF; the O_2_ interacts with the (101) facet of the pristine BiOF (**c**) and the BiOF with the O vacancy (**d**). Reprinted from Ref. [42] with permission from Elsevier.

In addition to dye degradation, OVs endow BiOF with outstanding capability toward perfluorooctanoic acid (PFOA) photodegradation. Wang and coworkers tuned the surface O vacancy concentration of (101) facet BiOF nanosheets by changing ethylene glycol dosage, and the optimized 50% EG-BiOF sample completely degrades PFOA within six hours under UV irradiation with a TOC removal efficiency of 56.8% [42]. As shown in Figure 6, EPR spectra show a progressive increase in the characteristic g ≈ 2.001 signal with EG content, confirming the rise in O vacancy concentration; O 1*s* XPS spectra quantify the relative vacancy content reaching approximately 17% for the optimal sample; and DFT adsorption models reveal that OVs raise the O_2_ adsorption energy on the (101) facet from −0.35 eV to −0.51 eV. Energy barrier calculations further confirm that photoelectrons preferentially attack the α-C-F bond with a barrier of only 4.56 kcal mol^−1^. Notably, photocurrent and fluorescence lifetime tests uncover a non-monotonic correlation between vacancy content and catalytic activity: moderate vacancies (about 17%) optimize carrier dynamics, while excessive vacancies turn into carrier recombination centers. This non-monotonic behavior, however, has not been reproduced by DFT calculations, which typically assume dilute defect limits and do not account for defect–defect interactions at higher concentrations. Moreover, the calculations were performed using GGA-PBE without SOC corrections, potentially affecting the accuracy of the reported barrier heights.

To elaborate on this irregular tendency, Ming Ren’s group synthesized a series of BiOF derivatives, including (BiO_0.51_F_1.98_), BiO_0.67_F_1.66_, Bi_7_O_5_F_11_ and BiOF, with varied O/F stoichiometry by adjusting hydrothermal solvents including acetic acid–water and tetrahydrofuran–water mixtures [67]. Unexpectedly, decreasing O/F ratio reduces O vacancy concentration verified by weakened EPR signals, yet the degradation activity toward methylene blue (MB) and RhB gradually rises, and BiO_0.51_F_1.98_ with the lowest O/F ratio achieves the optimal performance. Combined DFT and experimental characterizations explain this counterintuitive performance trend; higher fluorine content reduces oxygen vacancy density, yet it intensifies the IEF to boost carrier separation efficiency, accompanied by downward-shifted band edges that enhance the oxidation capability of valence band holes. EIS results further confirm that BiO_0.51_F_1.98_ exhibits the minimum interfacial charge transfer resistance. This work provides a crucial counterexample to the often-assumed positive correlation between OV concentration and activity. Nevertheless, the DFT calculations were limited to band structures of Bi_2_O_3_, BiOF, and BiF_3_ end-members, without directly modeling the non-stoichiometric BiO_0.51_F_1.98_ or BiO_0.67_F_1.66_ phases. Direct modeling of these complex structures would be valuable to confirm whether IEF enhancement or band edge shifts are indeed the dominant factors. Overall, this result reveals that catalytic performance cannot be determined solely by OV concentration, and comprehensive evaluation combining band structure and carrier kinetics is essential. A significant unresolved question remains: whether the optimal OVs concentration in Wang’s work (about 17%) and the OVs independent activity in Ren’s work can be reconciled within a unified framework. This requires systematic DFT studies that simultaneously consider vacancy thermodynamics, band edge positions, and charge transport across a wide composition range.

Beyond organic pollutant elimination, OVs also play critical roles in CO_2_ photoreduction and modified BiOF composite systems. In Ren’s systematic investigation on oxygen-vacancy-mediated CO_2_ photoreduction over BiOX (X = F, Cl, Br, I), positron annihilation lifetime spectroscopy identifies BiOBr with the highest O vacancy concentration and optimal CO production yield [68]. Notably, although BiOF exhibits the highest CO_2_ adsorption capacity among the BiOX series (0.4 cm^3^/g vs. 0.1 cm^3^/g for BiOBr), its photoreduction activity remains the lowest, suggesting that strong adsorption alone is insufficient for high catalytic performance and that factors such as band edge positions and surface reaction barriers play decisive roles. In composite systems, OVs in BiOF_x_Br_1−x_ solid solutions and S-scheme BiOBr/BiOF_0.7_I_0.3_ heterojunctions contribute to enhanced pollutant degradation and CO_2_ conversion through synergistic effects with band engineering and redox mediators [43,62,69]. Detailed mechanistic pathways, including the OVs-mediated cyclic CO_2_ reduction and the identification of reactive species, are discussed in Section 6.

Three intrinsic vacancies exert varied effects on BiOF. Bi vacancies drastically narrow the bandgap and improve conductivity, which is suitable for lithium-ion cathodes but rarely studied for photocatalysis. F vacancies only marginally reduce the bandgap and impair the built-in electric field, with limited practical photocatalytic value. OVs are the most effective defects, introducing intermediate energy levels, extending light absorption, and serving as active sites, though their concentration must be carefully optimized to avoid carrier recombination. Notably, the claimed effects of Bi and F vacancies require further computational validation using hybrid functionals with SOC to confirm defect level positions and electrostatic potential calculations to quantify the impact on the internal electric field. Table 4 summarizes the key DFT studies on BiOF defect engineering.

Table 5 summarizes the experimental and DFT findings on intrinsic defects in BiOF, including OVs, bismuth vacancies, and fluorine vacancies. OVs have been confirmed experimentally, and DFT predicts their formation energy, reduced bandgap, and enhanced adsorption and photocatalytic activity on specific facets, though the calculated bandgap remains lower than the experimental value, requiring higher-level corrections. In contrast, bismuth and fluorine vacancies have only been theoretically predicted and lack experimental validation. This Table highlights both the qualitative agreement and quantitative discrepancies between theory and experiment for OVs, while also pointing out research gaps for other defect types, providing a useful reference for future defect engineering studies.

## 4. Element Doping Optimization of the Electronic Structure, Light Response and Carrier Dynamics of BiOF

Doping constitutes an effective strategy to modulate the band structure of BiOF, broaden its light-response range, and improve photocatalytic performance [70]. According to dopant species, doping is classified into cation doping and anion doping. Despite shared underlying mechanisms as well as intrinsic discrepancies between the two categories, both approaches introduce impurity energy levels, narrow the bandgap, or optimize carrier dynamics without compromising the outstanding electrical insulation and structural stability of pristine BiOF. Cation doping serves as an important strategy to modulate BiOF electronic structure, which is classified into main-group metal doping, rare-earth doping, and isovalent cation doping based on dopant VBM.

**Figure 7:**
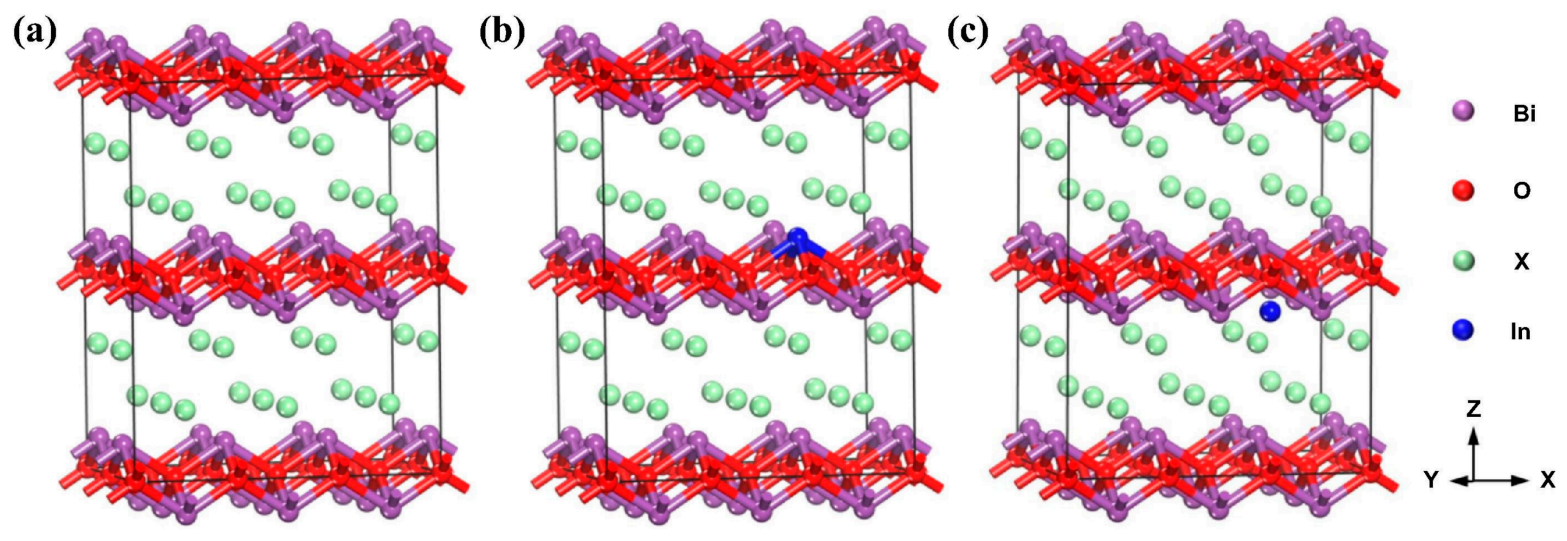
The optimized structures for (**a**) pure, (**b**) SubIn-doped BiOXs (X = F, Cl, Br, and I), and (**c**) IntIn-doped BiOXs (X = F, Cl, Br, and I). Reprinted from Ref [44] with permission from Elsevier.

First, Li et al. [44] used DFT to explore In^3+^ substituting Bi^3+^ in BiOF. Figure 7 illustrates the three structural models investigated by Li et al., including pristine BiOF, SubIn-doped (In substituting Bi), and IntIn-doped (In interstitial) models. In-doping lowers the CBM by ~0.3 eV while the VBM edge remains nearly unchanged, narrowing the bandgap from 3.1 eV to 2.7 eV without introducing in-gap localized defect states. Such band configuration avoids carrier trapping at defect sites and maintains BiOF intrinsic insulation and structural stability while improving visible-light utilization. However, several methodological concerns must be raised. First, SOC is not included in the original calculations, which may affect the predicted CBM shift. Second, the authors did not apply DFT + *U* or hybrid functionals despite acknowledging their necessity; PBE typically misplaces defect levels, and HSE06 + SOC verification is required to confirm the absence of in-gap states. Third, the study is purely theoretical and lacks experimental validation. Unlike In-doping, which preserves the direct-gap character, the rare-earth doping discussed below introduces 4*f* impurity levels that can switch the bandgap from direct to indirect, representing a fundamentally different modulation mechanism.

Second, rare-earth cation doping introduces 4*f*-derived mid-gap impurity levels to regulate band structure and photocatalytic performance. As reported by Wang et al. [70], Sm^3+^ doping introduces Sm 4*f* intermediate states inside the forbidden zone, narrowing the bandgap from 3.197 eV to 3.045 eV and transforming the direct bandgap into an indirect one. The 11 mol% Sm^3+^-doped sample achieves a RhB degradation rate constant of 0.0213 min^−1^, 2.7 times higher than pristine BiOF. For Eu^3+^ doping [71], Eu 4*f* impurity bands (~0.33 eV width) form within the bandgap, and the Eu^3+ 5^D_0_ level sits 0.28 eV below the CBM of BiOF, facilitating electron transfer from the CBM to Eu^3+^ and producing characteristic orange-red luminescence. Moreover, Er^3+^/Yb^3+^ co-doping [72] turns BiOF into an indirect semiconductor with a narrowed bandgap; Yb^3+^ harvests 980 nm near-infrared light and transfers energy to Er^3+^ for up-conversion visible emission. The optimal 2 mol% Yb^3+^ co-doped sample achieves 95.2% RhB removal under visible light and 19.9% under NIR irradiation, whereas bare BiOF shows almost no NIR activity. However, the computational treatment of rare-earth 4*f* electrons requires caution. Standard GGA-PBE, employed in all three studies, is known to inadequately describe localized 4*f* states without Hubbard *U* or hybrid-functional corrections. None of the cited works applied DFT + *U*, leaving the energetic positions of the 4*f*-derived impurity levels uncertain. SOC, which affects both Bi 6*p* and rare-earth 5*d* states, was also omitted in these calculations. These methodological limitations should be considered when interpreting the predicted bandgap narrowing and the proposed charge transfer pathways.

**Figure 8:**
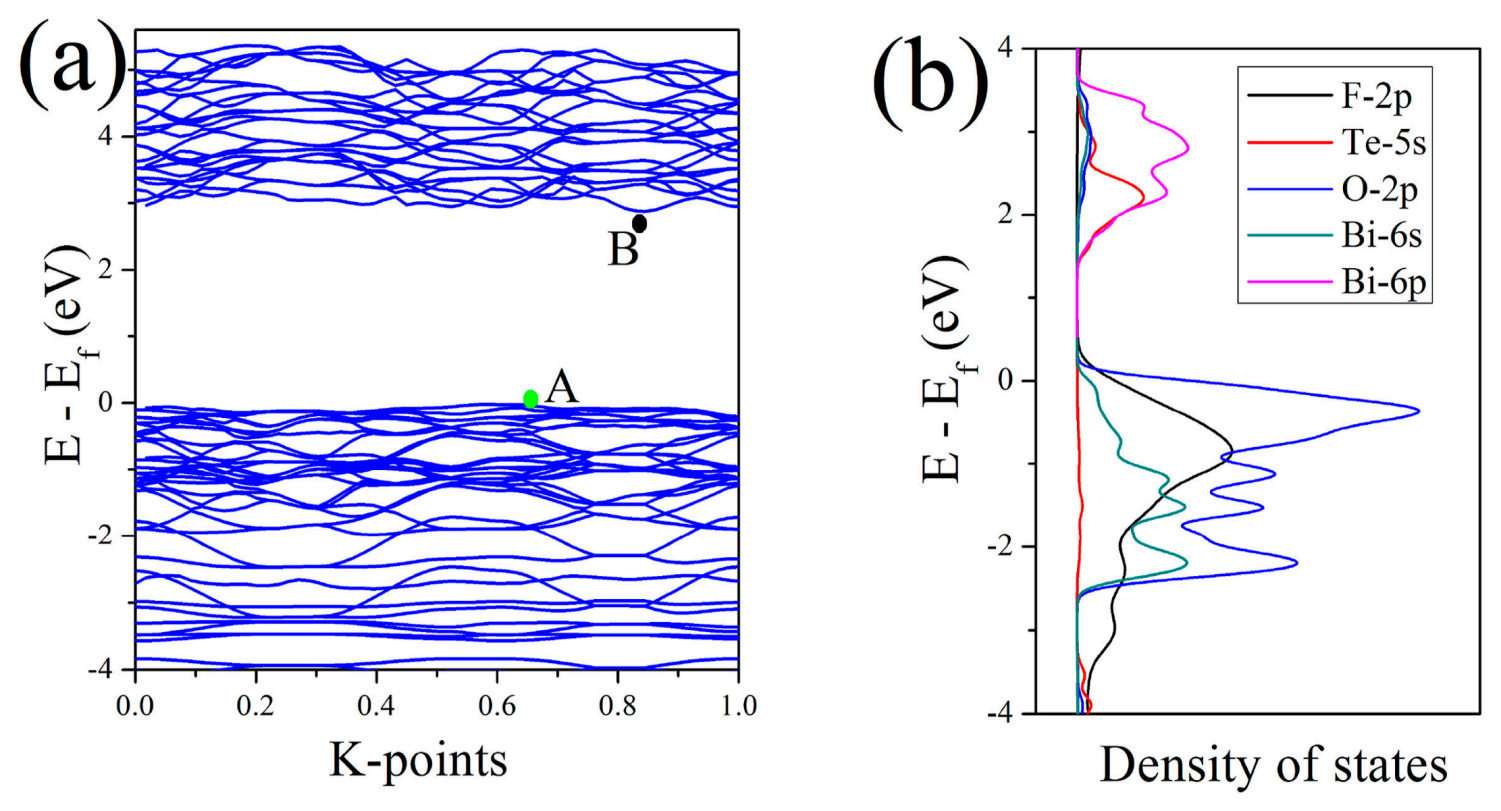
(**a**) Band diagram representation between −4 eV and 6 eV, and (**b**) the calculated partial density of states of BiTe_3_O_7_F. Reprinted from Ref [74] with permission from Elsevier.

Third, isovalent doping and off-stoichiometric cation regulation expanded BiOF application toward solid electrolyte and tunable band engineering. Ning et al. [73] substituted lattice Bi^3+^ with isovalent Ga^3+^, Al^3+^, In^3+^ and Yb^3+^ for fluoride solid electrolyte. The bandgap barely changes (2.86–3.37 eV vs. pristine 3.11 eV), yet dopant-induced lattice distortion damages regular Bi–F pyramidal coordination, weakens interlayer constraint over F^−^ and reduces the F^−^ diffusion barrier from 0.60 eV to 0.29–0.43 eV. Consequently, room-temperature ionic conductivity rises to 1.57 × 10^−2^–1.8 × 10^−2^ S/cm, satisfying the threshold for a practical solid electrolyte. Differently, Cai et al. [74] modulated Bi^3+^/Te^4+^ stoichiometry in BiTe_3_O_7_F for continuous bandgap tuning; excess Te^4+^ shrinks the lattice and widens the bandgap with absorption blueshift, whereas surplus Bi^3+^ expands the unit cell and narrows the bandgap for red-shifted absorption. Figure 8 shows the DFT band structure (a) and density of states (b) of BiTe_3_O_7_F, revealing a direct bandgap of about 2.92 eV, with the valence band maximum contributed by Bi 6*p*, O 2*p* and F 2*p* and the conduction band minimum by Bi 6*p* and Te 5*s*. The Te-rich sample (x = 0.12) degrades 96% RhB within 150 min, demonstrating the high flexibility of stoichiometry-based band engineering.

**Figure 9:**
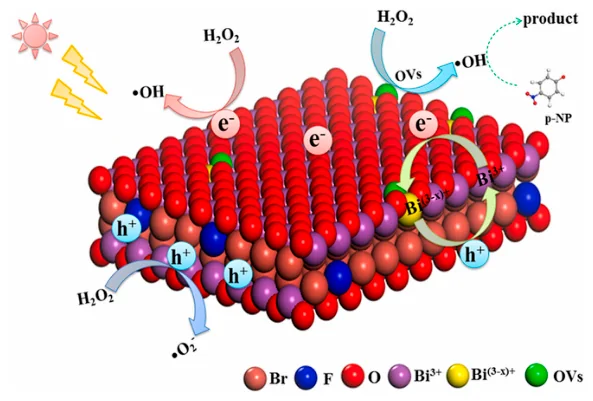
The possible photocatalytic mechanism of BiOF_x_Br_1−x_. Reprinted from Ref. [69] with permission from Elsevier.

Anion doping modulates BiOF band edges and surface defects effectively. Zhao et al. [69] prepared F^−^ partially substituted BiOF_x_Br_1−x_ solid solutions at ambient temperature. These solid solutions inherit the typical Sillén-phase alternating stacking structure, as illustrated in Figure 9, which establishes unique two-dimensional charge transport pathways and provides a natural lattice platform for anion substitution. Leveraging this structural advantage, DFT results confirm F substitution lowers CBM from −0.62 eV to −0.89 eV, strengthening the reduction potential for converting H_2_O_2_ into ·OH. Positron annihilation lifetime spectroscopy (PAL) and XPS confirm F/Br substitution introduces abundant OVs and Bi^3+^/Bi^(3−x) +^ redox sites; OVs content rises from 14.0% (BiOF_0.1_Br_0.9_) to 27.3% (BiOF_0.7_Br_0.3_). Benefiting from the above modifications, BF7 (BiOF_0.7_Br_0.3_) removes 96.1% of p-nitrophenol in 60 min, with an apparent rate constant of 0.045 min^−1^, 15-fold that of pristine BiOBr. Bai et al. [43] realized I^−^ substitution of F^−^ to tailor the BiOF electronic structure. Optimized BiOF_0.7_I_0.3_ (bandgap = 1.78 eV, CBM = −0.75 eV) matches BiOBr to construct high-efficiency S-scheme heterojunctions. The composite yields CO at 199.2 μmol·g^−1^·h^−1^, 10 times higher than pure BiOBr. Ren et al. [67] regulated BiOF O/F stoichiometry via solvent-controlled hydrothermal synthesis to obtain BiO_0.51_F_1.98_, BiO_0.67_F_1.66_, and Bi_7_O_5_F_11_and standard BiOF. A reduced O/F ratio weakens the OV EPR signal and lowers OV concentration but improves RhB/MB degradation, with BiO_0.51_F_1.98_ showing the best activity. DFT calculations on the Bi_2_O_3_, BiOF and BiF_3_ end-members, combined with experimental characterization, led Ren et al. to propose that higher F content may strengthen the interlayer built-in electric field, while EIS data corroborate the minimum charge-transfer resistance of BiO_0.51_F_1.98_. However, the quantitative reliability of the reported CBM shifts and band-edge positions should be treated with caution, as all calculations were performed at the GGA-PBE level without SOC corrections; the absolute band edge values may shift by several tenths of an eV when hybrid functionals with SOC are employed, as discussed in Section 3. Furthermore, the claimed enhancement of the IEF in Ren’s work lacks direct electrostatic potential calculations, which would be necessary to quantitatively confirm this interpretation.

A cross-comparison across the various doping schemes reveals two key trends. First, for bandgap narrowing, main-group metal dopants such as In^3+^ can achieve a reduction without introducing mid-gap trap states, while rare-earth dopants achieve similar narrowing but inevitably introduce localized 4*f* levels, making them more suitable for up-conversion or luminescence applications at the cost of potential carrier trapping. Second, for electrical conductivity, isovalent doping deliberately induces lattice distortion to reduce ion diffusion barriers, whereas the other doping types aim primarily to optimize photocatalytic activity. Third, anion doping offers a distinct advantage by simultaneously modifying surface defects and band edge positions, resulting in enhanced surface reactivity beyond what cation doping alone can achieve. This distinction makes anion doping particularly attractive for surface-sensitive reactions such as pollutant degradation and CO_2_ reduction. Importantly, regardless of the doping strategy, a common limitation across most studies is the insufficient use of hybrid functionals and spin–orbit coupling, leaving the predicted impurity levels and band-edge shifts quantitatively uncertain.

Complementary in functional mechanisms, both cation and anion doping should avoid generating deep-level recombination centers or excessive lattice damage. Future work will combine high-throughput DFT calculations and machine learning to rapidly screen optimal dopant species and doping dosages, and to exploit the synergistic effects of cation–anion co-doping for advancing the practical application of BiOF-based materials in photocatalysis and electrochemical energy storage.

In summary, elemental doping serves as a versatile strategy to tailor the band structure, extend the light-response range, and optimize carrier kinetics of BiOF. Cation doping primarily modulates photocatalytic performance by introducing impurity states, narrowing bandgaps, or altering bandgap character, whereas anion doping focuses more on band edge manipulation and surface defect engineering.

To facilitate a direct quantitative comparison between DFT predictions and experimental observations across the doping systems discussed above, Table 6 summarizes the key theoretical and experimental results for each doping strategy, along with the major computational limitations that remain to be addressed.

Table 7 summarizes the key research on BiOF doping modification, covering doped elements, doping types, primary mechanisms, effects on electronic structure, and main performance characteristics.

## 5. Charge Transfer at the BiOF Heterojunction Interface, Band Alignment, and Control of Built-In Electric Field

**Figure 10:**
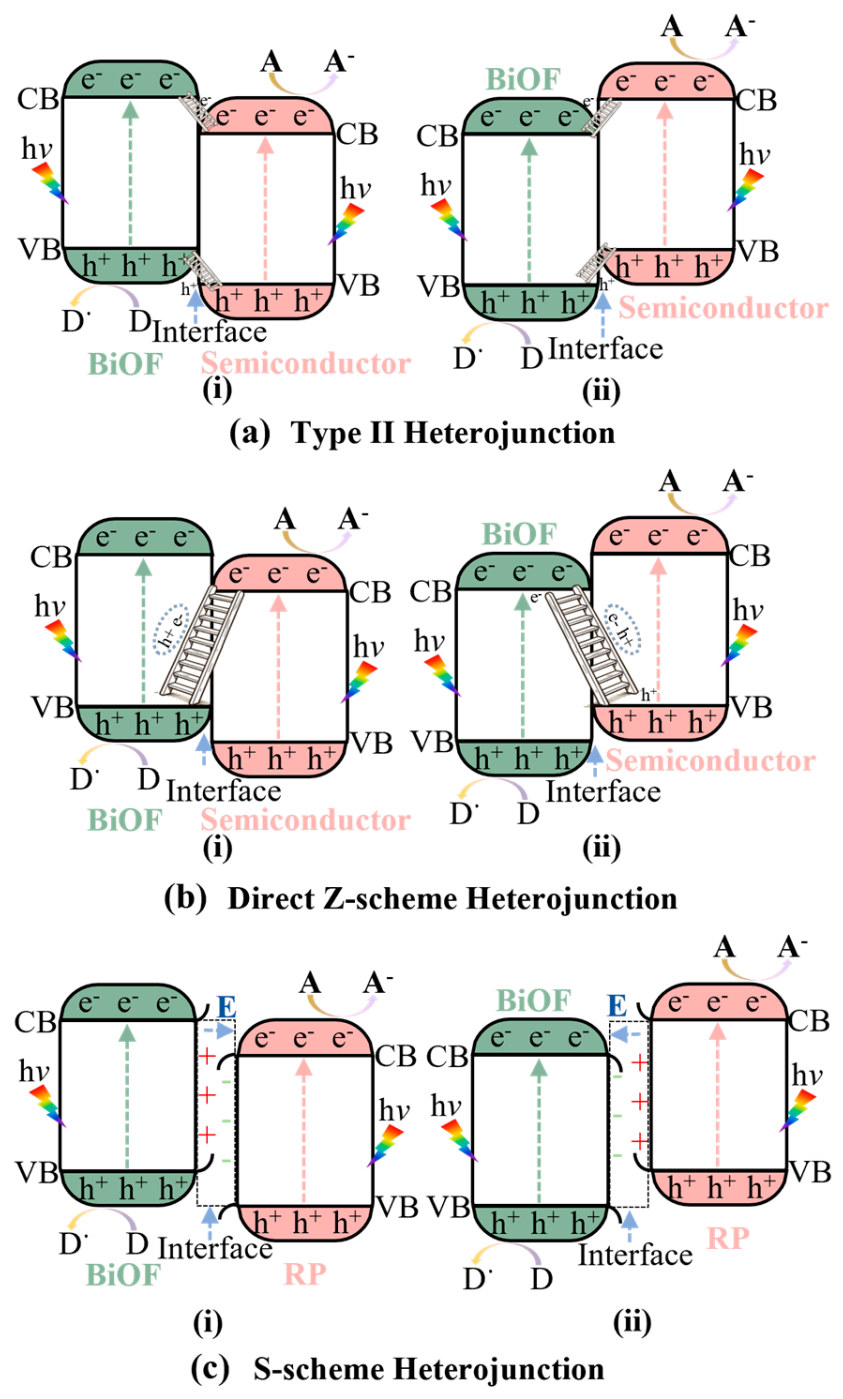
Four heterojunction mechanisms.

Single-component semiconductor materials generally struggle to simultaneously satisfy the requirements of broad-spectrum light harvesting, efficient charge separation, and robust redox capability [75]. It is difficult for single-component semiconductor photocatalysts to simultaneously realize broad-spectrum light absorption, efficient charge separation, and superior redox capability. Constructing BiOF-based heterojunctions by coupling with narrow-bandgap semiconductors provides an effective route to overcome these limitations [76]. As schematically depicted in Figure 10a–c, three representative heterostructure configurations, including Type-II, direct Z-scheme, and S-scheme, exhibit distinctly different interfacial charge migration behaviors. In Type-II, photogenerated electrons and holes migrate in opposite directions to the CBM and VBM of the two adjacent semiconductors, respectively. In the direct Z-scheme, low-energy carriers recombine at the intimate interface. In the S-scheme, the directional interfacial built-in electric field drives the selective recombination of useless carriers while preserving the high-energy electrons and holes on the respective components for subsequent redox reactions. Distinguishing these configurations experimentally is challenging; DFT calculations play an indispensable role here by predicting band-edge positions, work functions, and interfacial charge-density differences that ultimately determine the actual charge-transfer pathway. However, as discussed below, band alignment alone is insufficient to judge the heterojunction type, and formation energy and IEF direction, also derived from DFT, must be considered collectively to assess interfacial stability and carrier separation efficiency. According to band alignment configurations and interfacial charge-transfer pathways, several typical heterojunction architectures, including S-scheme and Z-scheme, as well as Type-II, have been elaborated in this work. DFT calculations play a pivotal role in predicting band edge positions, the direction of interfacial charge migration, built-in electric field intensity and the thermodynamic stability of constructed heterostructures.

**Figure 11:**
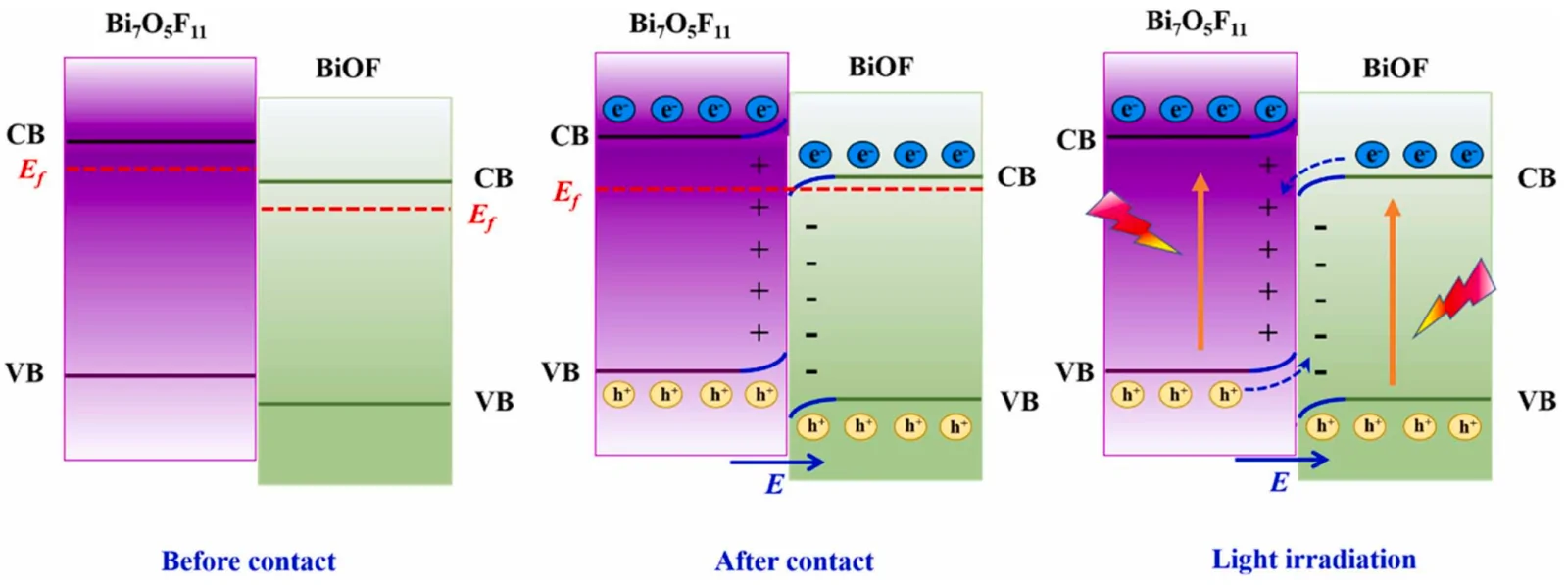
Schematic illustration of the photocatalytic mechanism for the BOF-2 composite under light irradiation, demonstrating the charge transfer mechanism of the S-scheme heterojunction. Reproduced from Ref. [78] with permission from Elsevier.

Among various configurations, S-scheme heterojunctions have attracted the most extensive attention for BiOF-based composites, owing to their ability to spatially separate carriers while retaining the most redox-active electrons and holes through interfacial recombination [77]. In the BiOBr/BiOF_0.7_I_0.3_ system [43], GGA-PBE calculations gave work functions of 5.06 eV for BiOBr and 4.73 eV for BiOF_0.7_I_0.3_. The work-function difference drives spontaneous electron transfer from BiOF_0.7_I_0.3_ to BiOBr in the dark, establishing an interfacial built-in electric field directed toward BiOBr. Upon illumination, this field, together with band bending, drives the interfacial recombination of CBM electrons of BiOBr with VBM holes of BiOF_0.7_I_0.3_, selectively preserving reductive electrons in BiOF_0.7_I_0.3_ and oxidative holes in BiOBr, which was subsequently confirmed by in situ XPS. To further optimize photocatalytic performance, dual OVs were rationally introduced into the composite. EPR and extended X-ray absorption fine structure (EXAFS) characterizations verify abundant surface OVs on BiOF_0.7_I_0.3_. These vacancy sites not only serve as active centers for CO_2_ adsorption and activation, with remarkably enhanced adsorption energy confirmed by DFT simulations, but also construct dynamic electron mediators coupled with the I^−^/I^0^ redox pair to accelerate interfacial charge transportation. As a result, the optimized S-scheme heterojunction delivers CO and CH_4_ evolution rates of 199.2 and 8.3 μmol·g^−1^·h^−1^, which are 10.0- and 6.7-fold higher than those of pristine BiOBr, respectively. However, the DFT calculations in this work do not isolate the individual contributions of OVs versus the I^−^/I^0^ pair to the overall activity enhancement; distinguishing these effects would require further systematic computational studies with controlled defect concentrations. Similarly, Wang et al. [78] constructed a Bi_7_O_5_F_11_/BiOF S-scheme heterojunction. DFT calculations determined their band edges: Bi_7_O_5_F_11_ has a CBM of −1.15 eV and VBM of 2.38 eV, while BiOF shows a CBM of −0.47 eV and VBM of 2.83 eV. The work-function difference drives spontaneous electron transfer from Bi_7_O_5_F_11_ to BiOF upon contact, balancing their Fermi levels and generating an interfacial built-in electric field with opposite band bending (Figure 11). Under UV irradiation, the interfacial field drives CBM electrons of BiOF to recombine with VBM holes of Bi_7_O_5_F_11_ following the S-scheme pathway, leaving high-reduction-potential electrons in the CBM of Bi_7_O_5_F_11_ and high-oxidation-potential holes in the VBM of BiOF, where the former reduce O_2_ to ·O_2_^−^ and the latter oxidize PFOA, respectively. In situ XPS and KPFM experimentally verify this unique charge-transfer route. Benefiting from strengthened PFOA adsorption and accelerated carrier separation, the BOF-2 composite fully mineralizes 15 mg/L PFOA within 1 h under UV light, with a TOC removal efficiency of 53%. Notably, the GGA-PBE calculations in this work omit SOC, which may affect the quantitative accuracy of the reported band-edge positions; however, the S-scheme assignment is robustly supported by the combined in situ XPS and KPFM evidence.

**Figure 12:**
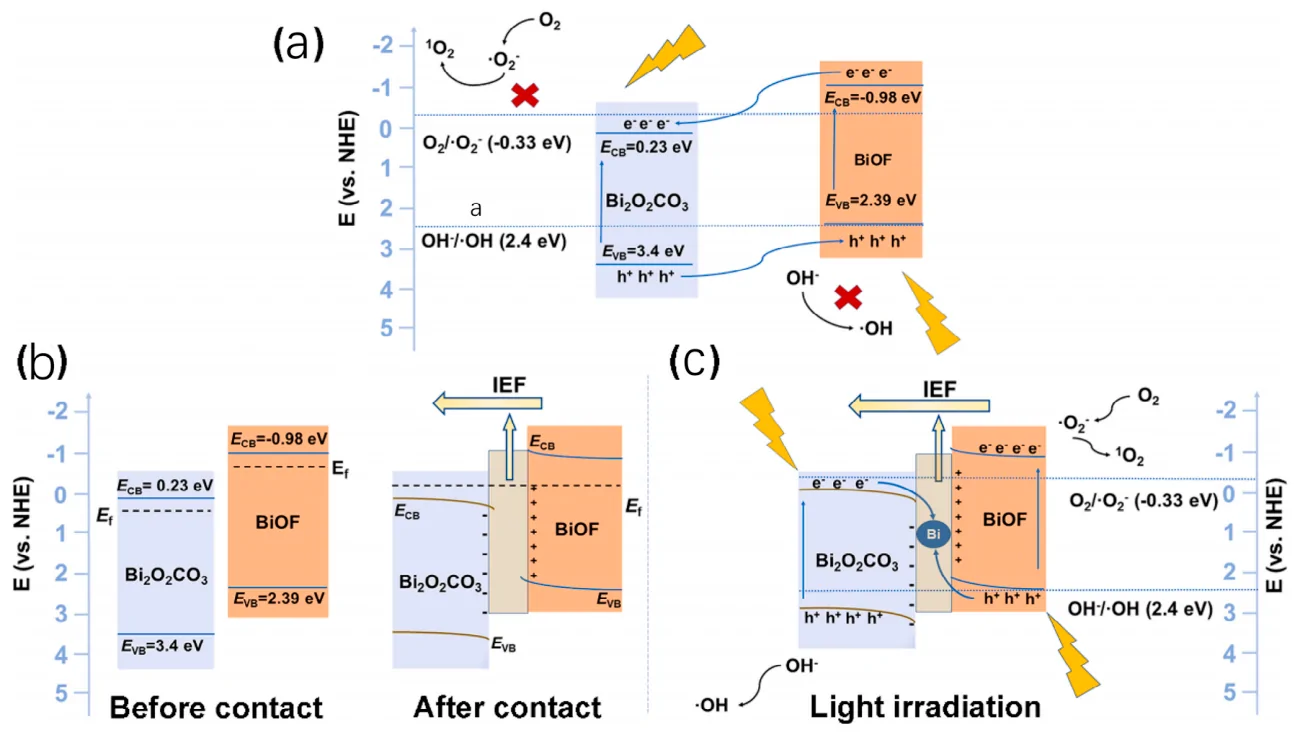
Heterojunction schematic of Bi/BiOF/BOC. Type-II Heterojunction (**a**), Z-scheme heterojunction without illumination (**b**), and Z-scheme heterojunction with illumination (**c**). Reproduced from Ref. [80] with permission from Elsevier.

Z-scheme heterojunctions generally require electron mediators or intimate solid–solid contact to sustain interfacial carrier recombination [79]. Ai and coworkers [80] constructed a ternary Bi/BiOF/Bi_2_O_2_CO_3_ Z-scheme heterostructure, wherein DFT calculations combined with high-resolution XPS analyses clarified the charge-transfer direction. After heterojunction construction, the binding energy of Bi 4*f* for Bi_2_O_2_CO_3_ shifts downward while that of BiOF increases, verifying spontaneous electron migration from BiOF toward Bi_2_O_2_CO_3_ and the concomitant formation of an interfacial built-in electric field oriented from BiOF to Bi_2_O_2_CO_3_. Figure 12 illustrates the Z-scheme charge transfer pathway at the heterojunction interface, with metallic Bi nanoparticles serving as electronic bridges. Metallic Bi nanoparticles serve dual functions: acting as electronic bridges to accelerate interfacial recombination along the Z-scheme pathway, and extending optical absorption and enhancing visible-light responsiveness via the surface plasmon resonance (SPR) effect of Bi. Under simulated solar illumination, the optimized photocatalyst achieves 99.8% degradation of ciprofloxacin within 60 min, with an apparent rate constant of 0.0649 min^−1^, which is 7 times and 3.5 times higher than those of pristine BiOF and bare Bi_2_O_2_CO_3_, respectively. Radical trapping experiments together with electron spin resonance (ESR) spectra confirm that h^+^ and ·O_2_^−^ dominate reactive species, consistent with the proposed Z-scheme charge-transfer mechanism. Another representative Z-scheme heterojunction is fabricated between ZnO and Yb^3+^/Er^3+^ co-doped Bi_3_Ti_2_O_8_F (denoted as BTOFYE). DFT computations reveal that BTOFYE possesses a lower work function (4.99 eV) relative to ZnO (5.24 eV); accordingly, electrons transfer spontaneously from BTOFYE to ZnO in the dark and establish a built-in electric field pointing from BTOFYE toward ZnO at the heterointerface [81]. The constructed internal electric field not only facilitates spatial separation of photogenerated carriers but also prominently boosts up-conversion luminescence of Er^3+^; the corresponding fluorescence lifetime increases from 85 μs to 123 μs, demonstrating a suppressed nonradiative transition induced by an interfacial electric field. Benefiting from the up-conversion effect, BTOFYE converts incident 980 nm NIR photons into visible emissions centered at 529, 546, and 662 nm, which are further captured by adjacent ZnO and hence extend photocatalysis toward the NIR region. After 120 min of irradiation, the Z-scheme composite attains 64% ciprofloxacin degradation, exhibiting 2.3-fold and 4.0-fold higher catalytic efficiency compared with single-phase BTOFYE and ZnO, respectively.

A cross-comparison of the two Z-scheme systems reveals that both rely on work-function-driven built-in fields to enable spatial charge separation, yet they differ in their auxiliary mechanisms: the Bi-mediated SPR effect in the former primarily enhances visible-light harvesting, whereas the up-conversion mechanism in the latter specifically enables NIR-driven photocatalysis. Notably, in both studies, DFT calculations serve a supportive rather than predictive role, in that they rationalize experimentally observed charge-transfer directions via work-function or XPS analyses rather than independently predicting novel configurations. Furthermore, the reported work functions were obtained at the GGA-PBE level without SOC corrections; hybrid-functional calculations would be needed to quantitatively validate the built-in field strengths.

**Figure 13:**
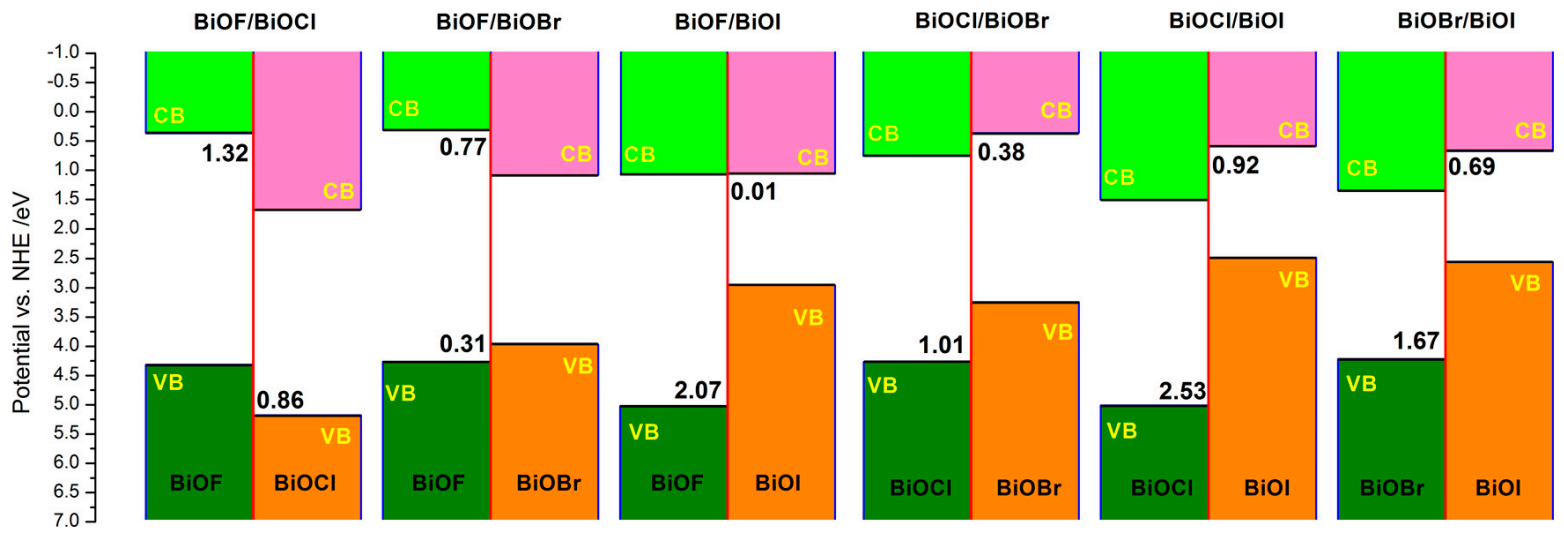
Calculated energy band positions of BiOX/BiOY (X, Y = F, Cl, Br, I) heterostructures. Reproduced from Ref [82]. Available under a CC-BY 4.0 license. Copyright 2023 Zhang et al.

Type-II heterojunctions rely on staggered band alignment to spatially separate photogenerated electrons and holes and suppress carrier recombination. Nevertheless, theoretical calculations indicate that BiOF/BiOCl and BiOF/BiOI heterojunctions are theoretically categorized as Type-II configurations with staggered band arrangements that are favorable for spatial carrier separation [82]. Figure 13 presents the calculated band-edge positions of six BiOX/BiOY heterostructures, clearly identifying BiOF/BiOCl and BiOF/BiOI as Type-II and BiOF/BiOBr as Type-I. However, their practical photocatalytic applications are severely restricted by inherent structural limitations. Firstly, these heterostructures exhibit high interfacial formation energies of 50.76 meV·Å^−2^ and 121.35 meV·Å^−2^, respectively, which are substantially higher than those of conventional BiOX composites (e.g., merely 3.03 meV·Å^−2^ for BiOCl/BiOBr), demonstrating poor thermodynamic stability and hindering their stable synthesis via routine experimental routes. Secondly, electrostatic potential analysis shows that the built-in electric fields of both BiOF/BiOCl and BiOF/BiOI systems are directed from BiOY (BiOCl or BiOI) toward BiOF, inducing electron migration from BiOF to BiOY. Such charge transfer direction conflicts with the intrinsic working principle of valid Type-II heterojunctions, in which electrons should migrate toward BiOF with a more negative CBM. The abnormal electric field orientation fails to facilitate efficient charge separation and may even exacerbate photogenerated carrier recombination. Therefore, despite their theoretical Type-II band alignment, the unfavorable thermodynamic stability and mismatched interfacial electric field distribution make BiOF/BiOCl and BiOF/BiOI inferior photocatalytic systems, which are not preferred for the rational design of high-performance BiOF-based photocatalysts.

Type-I heterojunctions feature nested band alignment, in which both photogenerated electrons and holes accumulate within the narrower-bandgap component and thereby hamper spatial charge separation; accordingly, such configurations are generally unfavorable for photocatalytic applications. Theoretical calculations identify BiOI/BiOF as a typical Type-I heterostructure. HSE06 hybrid-functional computations demonstrate that both the CBM and VBM of BiOI fall inside the bandgap of BiOF, inducing the simultaneous migration of electrons and holes toward BiOI and accelerating undesirable carrier recombination [83]. The positive formation energy (+0.44 eV) of this composite reveals that thermodynamically spontaneous heterojunction formation is inaccessible and external energy input is mandatory, imposing severe restrictions on practical experimental synthesis. In another systematic DFT investigation covering six possible BiOX/BiOY (X,Y = F, Cl, Br, I) combinations, BiOF/BiOBr is also categorized into the Type-I family, where both carriers converge on BiOBr and degrade photocatalytic performance [82]. Notably, although BiOF/BiOCl and BiOF/BiOI are theoretically predicted to follow Type-II band alignment, the built-in electric field oriented from BiOY toward BiOF may fail to facilitate effective carrier separation. A prerequisite for valid Type-II behavior is divergent carrier migration toward the two respective semiconductors, whereas an oppositely oriented intrinsic electric field will hinder such spatial separation. Moreover, the formation energy of BiOF/BiOY heterojunctions rises markedly with increasing atomic number of Y: the value increases from 50.76 meV·Å^−2^ for BiOF/BiOCl up to 121.35 meV·Å^−2^ for BiOF/BiOI, implying that despite favorable band arrangement for I-containing Type-II composites, inferior interfacial stability impedes their experimental fabrication. These theoretical results highlight a critical design principle for BiOF-based heterostructures: heterojunction type cannot be judged merely from band edge alignment. Complementary calculations of work function determine built-in electric field direction, formation energy evaluates interfacial thermodynamic stability, and charge density difference characterization verifies the actual interfacial charge transfer route.

In summary, efficient charge separation in BiOF-based heterojunctions relies on rational band alignment and interfacial built-in electric fields. S-scheme and Z-scheme heterostructures separate photocarriers spatially via interfacial recombination, retaining high-activity electrons and holes for applications such as CO_2_ photoreduction and organic degradation [43,78,80,81]. Band alignment alone cannot define heterojunction types; work function, formation energy, and charge density difference are essential to determine electric field direction, interfacial stability, and charge transfer pathways. Type-I heterostructures are undesirable for photocatalysis as they trap carriers within narrow-bandgap semiconductors, which can be excluded by theoretical calculations [82,83]. A cross-system comparison across the six heterostructure types reveals that S-scheme heterojunctions exhibit the strongest DFT-experiment agreement, with multiple techniques (in situ XPS and KPFM) validating the predicted charge-transfer pathways [43,78]. In these cases, DFT plays a predictive role, establishing the internal electric field direction prior to experimental confirmation. For Z-scheme systems, DFT serves a more interpretive function, rationalizing observed enhancements post-synthesis [80,81]. For theoretical Type-II and Type-I heterostructures, DFT fulfills a screening role, excluding configurations with prohibitively high formation energies or unfavorable IEF directions [82,83]. A common limitation across all studies is the use of GGA-PBE without SOC, which may affect the quantitative accuracy of band-edge positions. Furthermore, BiOF-based heterojunctions generally exhibit higher interface formation energies than non-BiOF combinations [82], reflecting the strong interlayer ionic bonding imparted by fluorine and underscoring the need for carefully designed in situ synthesis routes.

Table 8 summarizes the key DFT studies on BiOF-based heterojunctions, covering heterojunction types, charge transfer mechanisms, modification strategies, reaction systems, and optimal photocatalytic performance. Future work may focus on multicomponent and gradient heterojunctions, as well as the synergy between interfaces, defects, and doping. Combined with high-throughput screening, these studies will promote the practical application of BiOF-based photocatalysts.

Table 9 summarizes six research papers on BiOF-based heterojunction photocatalysis based on DFT calculations. The core content of the Table includes heterojunction types, charge transfer mechanisms, modification strategies, application/reaction systems, and the best performance.

## 6. Reaction Mechanism

Based on the intrinsic electronic structure of BiOF and its composite materials, DFT calculations play a crucial role in unraveling photocatalytic reaction pathways, origins of reactive species, and critical energy barriers; however, the reliability of these mechanistic conclusions depend sensitively on computational protocols such as the choice of exchange-correlation functional, SOC, and van der Waals corrections, which can shift adsorption energies and barrier heights by several tenths of an eV and potentially alter the predicted preference among competing pathways. Depending on the target catalytic reactions, photocatalysis is categorized into hydrogen evolution reaction (HER), oxygen evolution reaction (OER), organic pollutant photodegradation, and photocatalytic CO_2_ reduction. Rather than merely enumerating individual DFT results for these four systems, this section systematically reviews recent advances and applications of DFT simulations in mechanistic exploration with an emphasis on cross-comparison of computational methodologies and consistency between theoretical predictions and experimental observations.

### 6.1. Hydrogen Evolution and Oxygen Evolution

The HER (Equation (1)) and OER (Equation (2)) constitute the two half-reactions of overall photocatalytic water splitting. According to the band alignment of BiOF reported in previous studies [32,78], its VBM is far more positive than the water oxidation potential, endowing h^+^ with sufficient thermodynamic driving force to trigger OER; by contrast, the CBM is only slightly more negative than the proton reduction potential, resulting in inadequate driving force for HER. However, it should be noted that these band-edge positions were obtained from GGA-PBE calculations without SOC; hybrid-functional calculations with SOC yield appreciably different absolute band energies, which may alter the thermodynamic assessment of both OER and HER. For OER, trapping experiments during RhB photodegradation over BiOF nanosheets verify that h^+^ act as the predominant reactive species, while negligible ·OH signals are detected via ESR measurement. Such evidence reveals that holes directly oxidize organic contaminants rather than producing ·OH through intermediate water oxidation [41,58]. In the Bi_7_O_5_F_11_ heterostructure, the VBM of BiOF locates at +2.83 V, whose robust hole oxidation capability thermodynamically supports OER, and related experiments further confirm h^+^ as the dominant active species [78]. As regards HER, pristine BiOF delivers inferior hydrogen evolution activity due to its unfavorable CBM position, whereas BiOF-based heterojunctions exhibit promising prospects. In the S-scheme BiOBr/BiOF_0.7_I_0.3_ heterostructure, abundant electrons accumulate on the CBM of BiOF_0.7_I_0.3_ at approximately −0.75 V, satisfying the thermodynamic prerequisite of HER [43]; for the In–MOF/BiOF composite, the CBM of BiOF shifts to −0.40 V, close to the standard HER potential [84]. In summary, intrinsic BiOF suffers from limited inherent HER activity, yet rational construction of S-scheme or Z-scheme heterojunctions enables simultaneous preservation of strongly reductive CBM electrons and highly oxidative VBM holes [43,81], rendering BiOF-based materials theoretically feasible for overall water splitting. Future research should prioritize DFT calculations focusing on the Gibbs free energy of adsorbed hydrogen ΔG_H_ and elementary energy barriers of OER, alongside the exploration of interfacial synergy introduced by cocatalyst modification. For such future studies, the use of hybrid functionals with SOC and explicit solvation models is strongly recommended, as GGA-PBE without SOC is inadequate for accurately determining the absolute band-edge positions and reaction energetics of BiOF systems:

(1) $$\begin{array}{c} 2H^{+}+2e^{-}\to H_{2},E^{0}=-0.41\;\mathrm{eV} \end{array}$$

(2) $$\begin{array}{c} 2H_{2}O\to O_{2}+4H^{+}+4e^{-},E^{0}=+1.23\;\mathrm{eV} \end{array}$$

### 6.2. Decomposition of Organic Matter

BiOF-based photocatalysts are widely used for photodegradation of various organic pollutants, including dyes (RhB, MB), phenolic compounds (p-NP), antibiotics (CIP, TC), and persistent organic pollutants (PFOA, PFAS). DFT calculations are crucial for revealing the OV modulation effect, doping mechanisms, active species origin, and microscopic degradation pathways of BiOF photocatalysts.

In terms of dye photodegradation, (101)-faceted BiOF nanoplates with abundant surface OVs possess slightly narrowed bandgaps and accelerated electron transfer to generate ·O_2_^−^. h^+^ and ·O_2_^−^ are the main active species, delivering a photocatalytic rate 7.9-fold higher than bulk BiOF [41]. By comparison, (002)-oriented BiOF photocatalysis relies solely on h^+^ [58]. This facet-dependent shift in reactive species originates from the higher OV density on the (101) surface, which facilitates electron trapping and subsequent O_2_ reduction to ·O_2_^−^, whereas the (002) surface lacks sufficient OV sites for this pathway. Rare-earth doping introduces 4*f* impurity states to narrow the bandgap and transform the band structure; the Sm^3+^/Sm^2+^ redox cycle promotes ·O_2_^−^ production for synergistic pollutant degradation [70]. Co-doped BiOF achieves NIR-to-visible up-conversion, with doping-induced 4*f* levels trapping electrons to enable efficient RhB degradation under visible and NIR light [72]. A common limitation of these DFT studies on rare-earth doping is the use of GGA-PBE without DFT + *U* or hybrid functionals for describing localized 4*f* electrons, leaving the energetic positions of impurity levels quantitatively uncertain. Te-doped nonstoichiometric BiOF presents tunable bandgaps, and Te-rich samples realize 96% RhB removal within 150 min via ·O_2_^−^-dominated reactions [74].

For phenolic pollutant treatment using F-substituted BiOF_x_Br_1−x_ solid solutions, fluorine substitution negatively shifts the conduction band to boost photoelectron-induced ·OH generation from H_2_O_2_. OVs confirmed by XPS and PAL further promote H_2_O_2_ decomposition to produce extra ·OH. ·OH acts as the dominant active radical, achieving 96.1% p-NP degradation within 60 min [69]. However, the proposed mechanism that CBM shift enhances H_2_O_2_ to ·OH conversion remains largely inferential: the original DFT calculations established the band-edge shift, but did not directly compute the adsorption/dissociation energetics of H_2_O_2_ on the F-substituted surface or the barrier for ·OH generation. Whether OVs or the CBM shift plays the dominant role in promoting ·OH production is therefore not quantitatively resolved by DFT alone.

**Figure 14:**
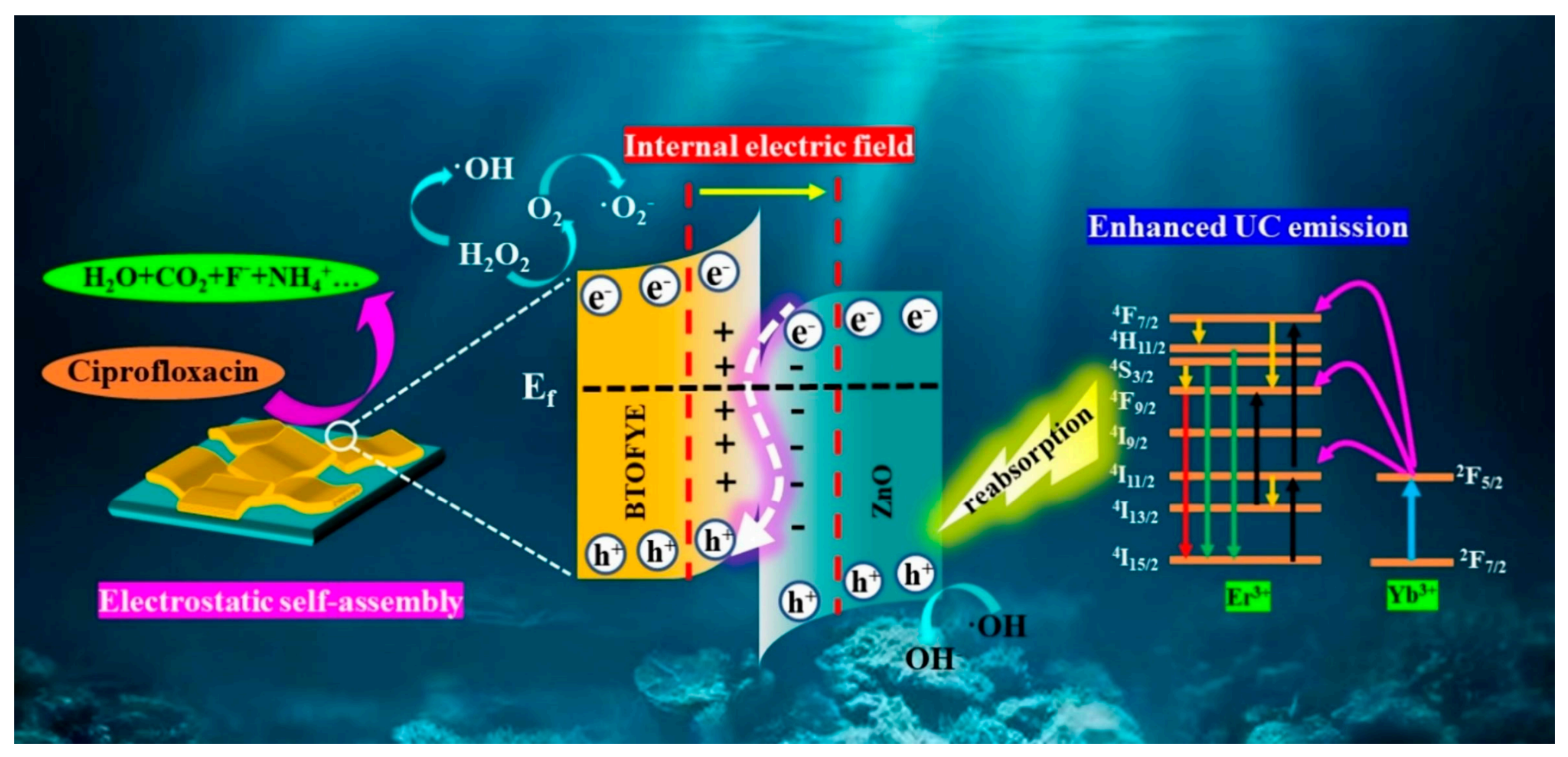
Photocatalytic enhancement mechanism of the ZnO/BTOFYE Z-scheme heterojunctions. Reproduced from Ref. [81] with permission from Elsevier.

In antibiotic degradation, Z-scheme Bi/BiOF/Bi_2_O_2_CO_3_ induces spontaneous interfacial electron migration verified by XPS. h^+^ and ·O_2_^−^ dominate oxidation, and LC-MS confirms two CIP degradation pathways, realizing 99.8% CIP removal in 60 min [80]. Another Z-scheme ZnO/Bi_3_Ti_2_O_8_F:Yb^3+^, Er^3+^ composite constructs a built-in electric field to enhance up-conversion luminescence and prolong fluorescence lifetime. Figure 14 illustrates the proposed Z-scheme charge transfer mechanism; the internal electric field facilitates spatial separation of photogenerated carriers, while Yb^3+^/Er^3+^ up-conversion converts NIR into visible emission, which is then absorbed by the composite to generate additional electron–hole pairs. This synergy yields 64% CIP degradation within 120 min via ·O_2_^−^ and ·OH [81]. This is used for DFT predictions and understanding the methodological limitations of these Z-scheme heterojunctions.

**Figure 15:**
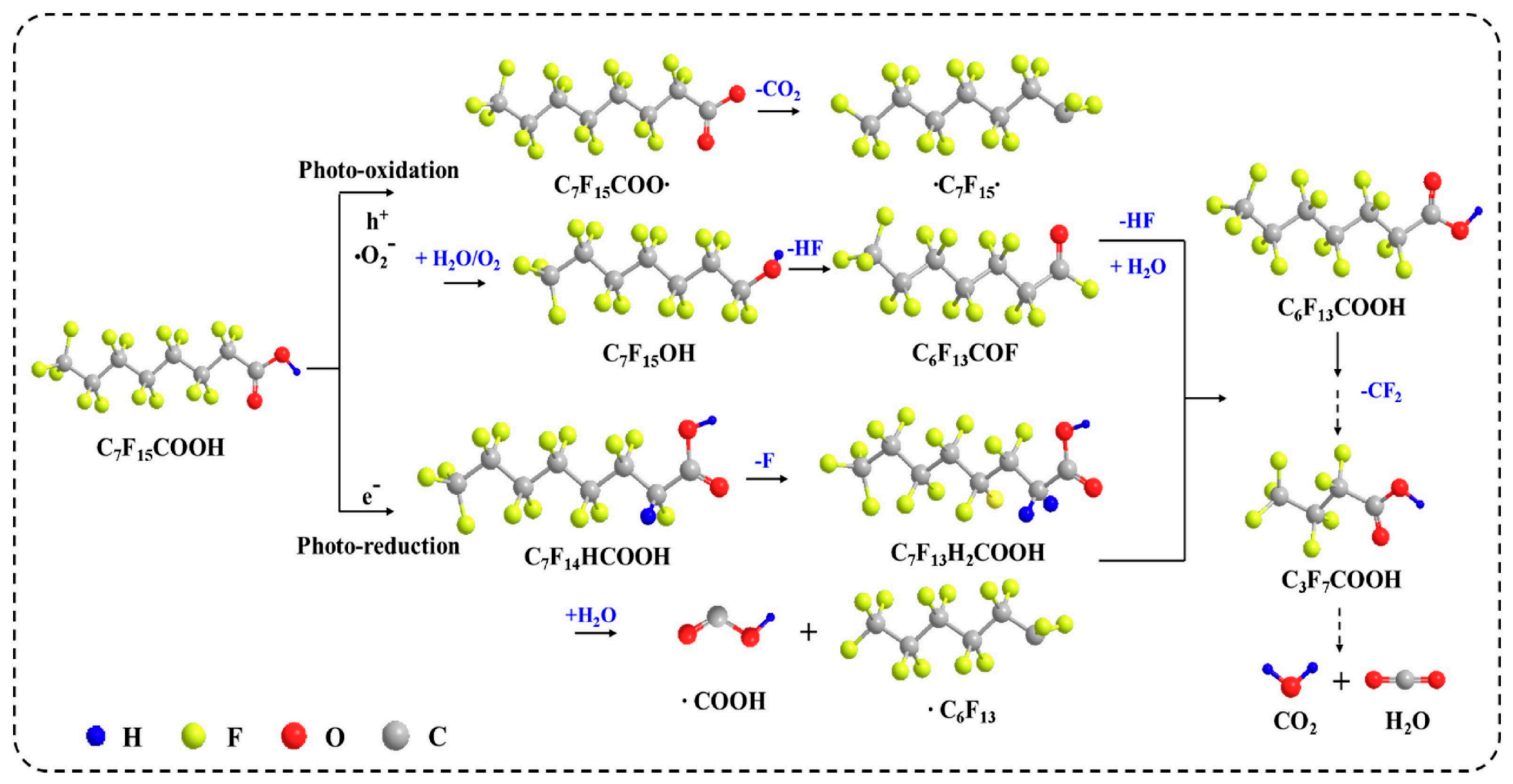
Degradation pathway of PFOA by 50% EG-BiOF photocatalyst during photocatalytic treatment. Reproduced from Ref. [42] with permission from Elsevier.

Regarding persistent organic pollutant elimination, DFT simulations on oxygen-vacancy-modified (101)-facet-exposed BiOF nanosheets provide comprehensive insights into PFOA mineralization, and Figure 15 schematically illustrates DFT-derived PFOA decomposition pathways together with energy barrier variations for each elementary step. Theoretical calculations indicate that the electron-initiated pathway is thermodynamically optimal: initial α-site C-F bond cleavage triggered by photoelectrons follows Equation (3), featuring a low energy barrier of 4.56 kcal·mol^−1^ and an exothermic reaction enthalpy of −5.93 kcal·mol^−1^. Afterwards, the generated intermediate C_6_F_13_FCHCOO^−^ undergoes hydrogen substitution as described in Equation (4), accompanied by an elevated barrier of 16.05 kcal·mol^−1^ and a reaction enthalpy of −2.44 kcal·mol^−1^. In the final step (Equation (5)), C_6_F_13_HCHCOO^−^ decomposes into short-chain perfluorocarbon radicals and CO_2_ with a barrier of 22.03 kcal·mol^−1^ and considerable heat release of −41.23 kcal·mol^−1^. By comparison, pathways dominated by ·O_2_^−^ and h^+^ display much higher barriers of 24.78 kcal·mol^−1^ and 18.98 kcal·mol^−1^, respectively. Accordingly, an electron-preferred α-C-F successive defluorination mechanism is proposed, realizing complete PFOA degradation and 56.8% TOC removal within six hours [42]. For the S-scheme Bi_7_O_5_F_11_/BiOF heterojunction, DFT calculations predict a PFOA adsorption energy of −1.33 eV, which is significantly more negative than that on pristine BiOF (−0.57 eV), and the composite enables complete PFOA degradation within 1 h [78]. DFT computes adsorption energies of four typical PFAS species over In-MOF/BiOF composites, with the order of PFOS > 6:2 Cl-PFESA > PFOA > HFPO-TA correlating with the observed degradation rate constants; PFOS is completely degraded within 90 min, with h^+^ and ·O_2_^−^ identified as the dominant reactive species [84]. Across these three DFT studies, a consistent strength is the quantitative prediction of adsorption energies and reaction barriers. However, the barrier heights in Yang et al.’s [42] work were obtained at the GGA-PBE level without SOC, which may affect the relative energetics among the competing pathways. For Wang et al. [78,84], DFT confirms enhanced adsorption and charge transfer, but the detailed degradation pathways and energy barriers for PFAS species remain uncomputed. In addition, comparative TC degradation measurements on four bismuth oxyhalides BiOX (X = F, Cl, Br, I) reveal that BiOBr delivers the highest photocatalytic activity owing to the smallest effective mass of photogenerated carriers, whereas BiOF exhibits inferior performance restricted by its widest bandgap and limited light harvesting [56].

In summary, DFT calculations have systematically uncovered the microscopic degradation mechanisms of various organic contaminants over BiOF-based photocatalysts from multiple perspectives, including band structure, defect energy levels, molecular adsorption energy, elementary reaction barriers, and interfacial charge migration, laying solid theoretical guidance for the rational design of high-performance photocatalytic materials:

(3) $$\begin{array}{c} C_{7}F_{15}COO^{-}+e^{-}\to TS1\to C_{6}F_{13}FCHCOO^{-} \end{array}$$

(4) $$\begin{array}{c} C_{6}F_{13}FCHCOO^{-}\to TS2\to C_{6}F_{13}HCHCOO^{-} \end{array}$$

(5) $$\begin{array}{c} C_{6}F_{13}HCHCOO^{-}\to TS3\to C_{6}F_{13}+CO_{2} \end{array}$$

### 6.3. Carbon Dioxide Reduction

**Figure 16:**
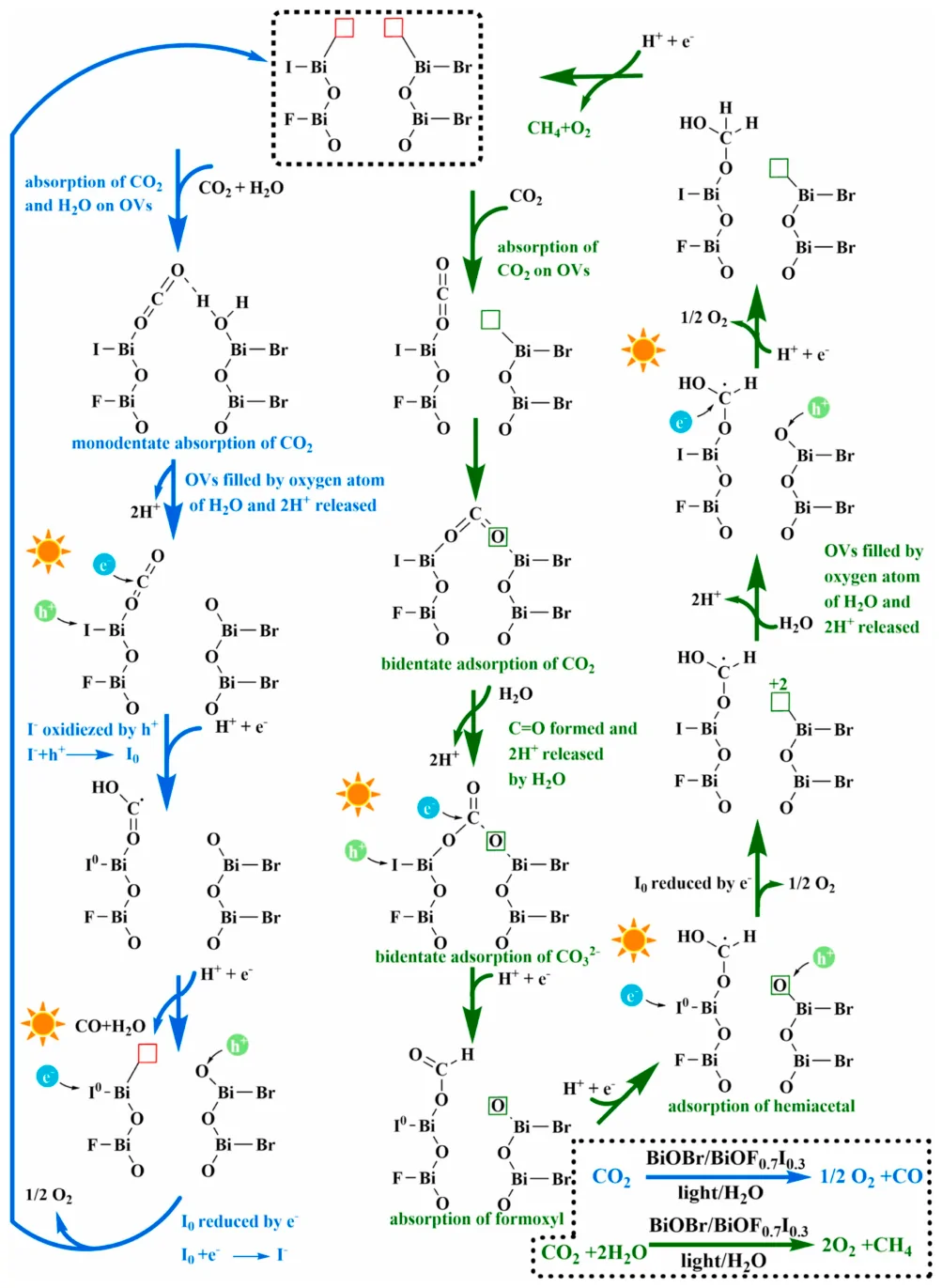
Proposed mechanism of CO_2_ photoreduction for BiOBr/BiOF_0.7_I_0.3_. Reproduced from Ref. [43] with permission from Elsevier.

Photocatalytic CO_2_ reduction is a multi-electron conversion process that transforms CO_2_ into carbon-based fuels such as CO, CH_4_, and CH_3_OH [85,86], and considerable progress has been achieved in S-scheme BiOF-based heterostructures toward this reaction. For the S-scheme BiOBr/BiOF_0.7_I_0.3_ composite [43], the photocatalytic mechanism and interfacial charge transfer pathway have been comprehensively characterized by DFT calculations combined with in situ XPS. As proposed in Figure 14 of the original work, CO_2_ molecules are adsorbed onto the dual oxygen vacancies in either monodentate (via one O atom) or bidentate (via two O atoms) configurations; these adsorbed CO_2_ species then interact with co-adsorbed H_2_O through hydrogen bonds to form critical COOH* intermediates. Under illumination, the I^−^/I^0^ redox couple undergoes cyclic oxidation–reduction, effectively suppressing electron–hole recombination. Subsequent proton-coupled electron transfer steps convert the intermediates into CHO and CH_3_O, and finally yield CO and CH_4_. This complete mechanistic pathway is schematically summarized in Figure 16.

Benefiting from such structural advantages, the heterostructure delivers a CO evolution rate of 199.2 μmol·g^−1^·h^−1^ and a CH_4_ yield of 8.3 μmol·g^−1^·h^−1^, which are 10.0-fold and 6.7-fold higher than those of pristine BiOBr, respectively. A systematic activity comparison among four BiOX (X = F, Cl, Br, I) photocatalysts for CO_2_ photoreduction has been performed [68]; DFT simulations demonstrate that OVs introduce mid-gap states and flatten the VBM to facilitate carrier separation. Positron annihilation lifetime spectroscopy confirms the highest O vacancy concentration in BiOBr, and electron density calculation reveals the largest electron population around lattice O atoms of BiOBr, which accelerates CO_2_ activation. Key intermediate species including m-CO_2_ (monodentate carbonate, 1480 cm^−1^), b-CO_2_ (bidentate carbonate 1280, 1363, 1549 cm^−1^) and COOH* (carboxyl intermediate 1312, 1597 cm^−1^) are captured via in situ FTIR, verifying the oxygen-vacancy-mediated CO_2_ reduction pathway; the O atom of adsorbed CO_2_ fills the original O vacancy site and turns into lattice oxygen, triggering distortion of adjacent Bi–O bonds; meanwhile, neighboring lattice oxygen is oxidized by h^+^ to regenerate fresh OVs and sustain cyclic catalysis. The photocatalytic activity follows the sequence BiOBr > BiOCl > BiOI > BiOF. Pristine BiOF exhibits the poorest performance owing to its widest bandgap, weakest CO_2_ adsorption affinity, and insufficient reduction capacity of CBM electrons.

In conclusion, DFT calculations have elucidated the underlying CO_2_ reduction mechanism of S-scheme BiOF composites from multiple dimensions, covering work function discrepancy, band alignment, vacancy adsorption energy, and intermediate identification. Nevertheless, intrinsic BiOF possesses a wide bandgap and unsatisfactory catalytic activity; rational heterostructure construction is indispensable to accomplish efficient photocatalytic CO_2_ conversion.

### 6.4. Critical Assessment of the Strengths, Limitations and Modification Strategies of BiOF

Based on the systematic discussion in the preceding sections, this subsection critically summarizes the intrinsic advantages and inherent shortcomings of BiOF, and evaluates the effectiveness and limitations of various modification strategies. The intrinsic advantages of BiOF are threefold. First, the alternating [Bi_2_O_2_]^2+^ and double F^−^ layers generate a built-in electric field along the c-axis that drives spatial charge separation, as experimentally validated by EIS and PL measurements [58]. Second, the VBM at +2.83 V vs. NHE endows holes with strong direct oxidation capability without relying on ·OH intermediates, while the ionic Bi–F bonds confer the highest elastic modulus and thermal stability among BiOX compounds. Third, the (101) facet possesses a lower oxygen vacancy formation energy than the (001) facet, making BiOF an ideal model system for synergistic facet and defect engineering.

However, several inherent limitations persist. The excessively wide bandgap (3.6–3.9 eV) experimentally [32] renders BiOF completely inactive under visible light, which constitutes approximately 45% of the solar spectrum. The CBM at approximately −0.47 V vs. NHE provides insufficient driving force for HER and CO_2_ reduction compared to BiOCl and BiOBr. Additionally, the interface formation energies of BiOF-based heterojunctions, for instance, 50.76 meV·Å^−2^ for BiOF/BiOCl, are substantially higher than non-BiOF combinations, such as BiOCl/BiOBr [78,82], constraining the synthesis window. Finally, the apparent agreement between GGA-PBE and experimental bandgaps is fortuitous, arising from error cancelation rather than physical accuracy, and the treatment of Bi semicore states and van der Waals corrections significantly affects band edge positions and defect levels, undermining the direct comparability of theoretical results across studies.

Various modification strategies exhibit different degrees of effectiveness in overcoming these limitations. Oxygen vacancy engineering is the most cost-effective approach for bandgap narrowing, which reduces the bandgap from 3.12 eV to 2.65 eV via Bi 6*p* mid-gap states. However, the non-monotonic relationship between OV concentration and activity, with 17% being optimal and higher concentrations converting vacancies into recombination centers [42], has not been reproduced by DFT calculations that typically assume the dilute defect limit. Bi and F vacancies are of limited relevance for photocatalysis due to the lack of experimental validation and high formation energy, respectively. Elemental doping offers differentiated pathways. In^3+^ substitution narrows the bandgap without introducing mid-gap trap states, representing a more desirable strategy, although HSE06 + SOC verification is needed. Rare-earth doping introduces 4*f* states for visible and NIR response but risks recombination centers due to the absence of DFT + *U* treatment for 4*f* electrons. Halogen substitution, such as Br and I, effectively modulates CBM positions; for example, F→Br shifts the CBM from −0.62 eV to −0.89 eV, while O/F stoichiometry tuning suggests the possibility of “fewer defects, higher activity” [67]. Among heterojunction strategies, S-scheme heterojunctions, such as BiOBr/BiOF_0.7_I_0.3_, most effectively overcome the insufficient reduction capability of BiOF, with CO_2_ reduction activity enhanced by a factor of 10. Z-scheme systems introduce SPR effects for broader light response. However, Type-II BiOF/BiOCl and BiOF/BiOI heterojunctions are practically infeasible due to unfavorable IEF direction and prohibitively high interface formation energies exceeding 50 meV·Å^−2^ [82]. Facet engineering selectively exposes the (101) high-activity facet, achieving a 7.9-fold enhancement in RhB degradation rate [41], but DFT has not yet quantified the exact difference in OV formation energy between the (101) and (001) facets.

In summary, OV engineering offers the most economical bandgap narrowing but requires careful concentration control. In^3+^ doping provides a trap-free tuning pathway. S-scheme heterojunctions most powerfully overcome the reduction capability deficit. Facet engineering naturally synergizes with defect engineering. Notably, the coupled effects of multiple strategies, such as OVs-modified S-scheme heterojunctions [43] or rare-earth-doped Z-scheme systems [81], often surpass linear superposition, suggesting that future design should shift from single-strategy optimization to multi-strategy integration. A critical open issue is that most DFT calculations for evaluating these strategies do not employ the HSE06 + SOC level, precluding a unified quantitative benchmark for comparing band edges, defect levels, and interfacial potentials across different strategies, thereby limiting the precision of theoretical guidance for experimental design.

Combining first-principles DFT calculations with experimental characterizations is an effective strategy to reveal the intrinsic structure–activity relationships of BiOF-based materials. Table 10 systematically summarizes representative BiOF-related DFT studies covering photocatalytic pollutant degradation, CO_2_ photoreduction, lithium-ion batteries, and electro-sorption applications. For each material system, we sort out the key parameters predicted by DFT, corresponding experimental performance, the consistency between theory and measurement, and the derived structure–property rules. Such comparison intuitively demonstrates the strengths and existing limitations of current DFT simulations for BiOF systems.

## 7. Summary and Outlook

This review summarizes DFT advances of BiOF and its modified systems covering crystal structure, intrinsic properties, defects, doping, heterojunctions and photocatalytic mechanisms. As the sole direct-bandgap member in the BiOX family, tetragonal BiOF has a VBM dominated by hybridized O 2*p*, F 2*p* and CBM governed by Bi 6*p*; high-electronegativity F creates a *c*-axis intrinsic built-in electric field from [Bi_2_O_2_]^2+^ toward F-layers to boost charge separation. Thermomechanical calculations show BiOF features the largest elastic modulus and Debye temperature yet low Vickers hardness originating from ionic Bi-F bonding, alongside an elevated Grüneisen parameter for larger thermal expansion. OVs introduce Bi 6*p*-related mid-gap states to narrow the bandgap and improve O_2_/CO_2_ activation; the (101) facet of BiOF with abundant surface vacancies outperforms the (001) bulk, while excessive vacancies turn into recombination centers, and Bi vacancies effectively narrow the bandgap for potential lithium battery cathode use. In dopant engineering, In^3+^ realizes trap-free bandgap narrowing, rare-earth doping introduces 4*f* impurity levels and changes bandgap type, and Er^3+^/Yb^3+^ co-doping achieves NIR response via up-conversion; halide substitution and O/F stoichiometry adjustment optimize band alignment and built-in field strength to promote ·OH generation. Among heterostructures, S/Z-scheme heterojunctions spatially separate carriers and reserve high redox potentials for efficient CO_2_ photoreduction and organic degradation, whereas Type-I and partial Type-II heterojunctions are restricted by unfavorable band matching or high formation energy. DFT also clarifies core reaction paths: vacancies accelerate superoxide formation, PFOA degradation preferentially proceeds via electron-driven α-site C-F cleavage, and intermediate signals confirm a vacancy-mediated CO_2_ reduction mechanism.

Despite these advances, several unresolved scientific challenges remain. The non-monotonic relationship between OV concentration and activity has been observed experimentally but not reproduced by DFT; the quantitative roles of OVs versus the I^−^/I^0^ redox pair in S-scheme heterojunctions have not been distinguished; PFAS degradation pathways beyond PFOA lack computed energy barriers; HER and OER elementary steps have not been systematically evaluated for any BiOF surface; and the selectivity between CO and CH_4_ in CO_2_ reduction remains unexplained. To tackle these challenges, high-accuracy calculations (HSE06, vdW correction, SOC, and BSE) should be adopted to establish a reliable computational benchmark. Non-adiabatic molecular dynamics can complement static DFT by simulating ultrafast carrier dynamics at defect sites and heterojunction interfaces. Machine learning, when integrated with high-throughput DFT, can accelerate the screening of dopants and heterojunction combinations, while Bayesian optimization can guide experimental synthesis toward optimal facet exposure and defect concentrations. Beyond conventional photocatalysis, BiOF shows potential in next-generation applications such as deep-ultraviolet optoelectronics, sodium/potassium-ion batteries, and integrated capture-and-degrade systems for per-fluorinated pollutants, though experimental validation and further DFT-guided optimization are required.

Tight integration of theory and experiment remains the overarching prerequisite for realizing these opportunities. DFT can predict synthesis conditions and spectral features to guide experiments, while experimental characterizations provide feedback to refine computational models. This reciprocal cycle, combined with emerging computational tools, will accelerate the rational design of high-performance BiOF-based materials for photocatalysis, optoelectronics, and energy-related technologies.

## Figures and Tables

**Table 1:** Summary of structural, electronic, and optical parameters for bulk BiOF.

Structural & Electronic Parameters	Value	Optical & Dielectric Parameters	Value
Crystal system/Space group	Tetragonal P4/nmm (PbFCl-type) [58]	Bandgap (GGA-PBE) (eV)	3.02–3.34 [33,59,61]
a (Å)	3.710–3.787 [32,33,59] (DFT) 3.748–3.760 (Expt.) [58]	Bandgap (HSE06) (eV)	4.18–4.47 [32]
c (Å)	6.122–6.293 [32,33,59] (DFT) 6.224–6.230 (Expt.) [58]	Bandgap (G_0_W_0_) (eV)	4.04 [59]
Bi–O bond length (Å)	2.278–2.312 (DFT) [61]	Bandgap (mBJ) (eV)	4.85–5.18 [33]
Bi–F bond length (Å)	2.75–2.794 (DFT) [61]	Bandgap (PBE + umBJ + SOC) (eV)	3.34–3.50 (PBE + umBJ + SOC) [33]
Bi effective charge (e)	+1.50 (Mulliken) [61] +1.65 (Löwdin) [55]	Experimental bandgap (eV)	3.64–3.93 [33]
O effective charge (e)	−0.84 (Mulliken) [61]	Bandgap type	Direct (PBE/HSE/GW) [33,59] Near-direct (LDA/PBEsol/WC) [33]
F effective charge (e)	−0.66 (Mulliken) [61]	BSE optical gap (eV)	~3.75 [59]
Cell volume V (Å^3^)	87.67 [61]	Exciton binding energy (eV)	~0.3 [59]
IP (eV)	8.23 (HSE06 + SOC) [32]	Static ε_1_(0) (xx/zz)	4.82/4.34 (PBE) [61] 3.80 (BSE) [59]
EA (eV)	3.87 (HSE06 + SOC) [32]	Electron effective mass (m_e_)	1.0 (in-plane, Z→R)/0.5 (out-of-plane, Z→Γ) (HSE06 + SOC) [32]
—	—	Hole effective mass (m_e_)	2.5→m_e_ (HSE06 + SOC) [32]

**Table 2:** Summary of facet-dependent properties of BiOF from DFT calculations and experimental characterizations.

Facet	Surface Energy (J/m^2^)	Adsorption Energy (eV)	Exposure Ratio	Dominant Reactive Species	Photocatalytic Rate (min^−1^)	Ref.
(001)	–	−3.16(for F^−^)	–	h^+^	–	[62,63]
(002)	3.11	–	75.4%	h^+^	0.02534	[58]
(101)	–	−3.91(for F^−^)	–	h^+^, ·O_2_^−^	0.098	[62,63]

**Table 3:** Comparison of pseudopotential and basis set combinations used in DFT calculations for BiOF and related BiOX systems.

Method	Bi Valence Configuration	vdW Correction	SOC	BiOF Bandgap (eV)	Advantages
PBE + USPP	6*s*^2^6*p*^3^ (5 e^−^)	No	No	3.12 [54]	Computationally efficient
PBE + NCPP	5*d*^10^6*s*^2^6*p*^3^(15 e^−^)	No	No	3.22 [54]	Bi 5*d* treated as valence
PBE + PAW + D3	5*d*^10^6*s*^2^6*p*^3^	DFT-D3	No	3.07 [64]	Includes vdW correction
HSE06 + PAW + D3 + SOC	5*d*^10^6*s*^2^6*p*^3^	DFT-D3	Yes	4.18 [32]	Highest accuracy; excellent agreement with experiment
PBE + umBJ + SOC (LAPW)	5*d*^10^6*s*^2^6*p*^3^	No	Yes	3.34 [33]	Good balance of accuracy and efficiency
PBE + mBJ + SOC (LAPW)	5*d*^10^6*s*^2^6*p*^3^	No	Yes	4.22 [33]	Improved gap over PBE

**Table 4:** Some of the main papers discussing the BiOF defect.

Ref.	Defect Type	Research Technique	Key Performance	Electron Transfer Mechanism	Other DFT Results
[62]	OVs	Hydrothermal synthesis; Electrochemical testing; XPS/EPR; DFT: VASP, GGA–PBE, 450 eV.	Under 0.9 V, BOF-C achieves a fluoride adsorption capacity of 24.14 mg·g^−1^, with separation factors of 6.585 (F^−^/Cl^−^), 6.978 (F^−^/Br^−^), and 8.244(F^−^/${NO}_{3}^{-}$).	Reduction potential triggers Bi^3+^ reduction and F^−^ release; oxidation reverses it. High (101) exposure lowers diffusion resistance (CV confirms diffusion control; EIS shows steep low–frequency slope).	The density of states was calculated separately for the (101) and (001) facets. Additionally, the adsorption energies of fluoride ions on these two distinct surfaces were obtained to allow a comparative analysis between them.
[41]	OVs	Samples were hydrothermally synthesized and characterized by photocatalytic tests, EPR, PL, and XPS. DFT calculations employed the CASTEP platform with the GGA-PBE functional and a plane–wave cutoff energy of 380 eV.	Under simulated solar illumination, BiOBr exhibits optimal CO and CH_4_ production rates of 21.6 and 1.2 μmol·g^−1^·h^−1^, respectively, with nearly 100% CO selectivity at high light intensity and an apparent quantum efficiency of about 1.26%.	OVs trap electrons, converting O_2_ to ·O_2_^−^, while holes oxidize RhB. Despite a high Rct (0.89 Ω), the sample exhibits the weakest PL and highest photocurrent, indicating effective carrier separation.	The surface free energies were calculated separately for the (002) and (101) facets of BiOF to compare the relative stability of these two exposed surfaces.
[42]	OVs	All samples were hydrothermally synthesized and characterized by photocatalytic evaluation, EPR, XPS, PL, and photoelectrochemical tests. DFT calculations used the VASP code with the GGA–PBE functional.	Under UV irradiation, 50% EG-BiOF completely degrades PFOA within 6 h with a rate constant of 0.36 h^−1^ (2.4× pristine BiOF), a TOC removal of 56.8%, and a defluorination ratio of 26%.	OVstrap electrons to reduce O_2_ to ·O_2_^−^, while holes oxidize PFOA. Optimal 17% vacancy content raises photocurrent 2.3× and extends PL lifetime to 16.64 ns (pristine: 5.45 ns). DFT shows electrons preferentially attack the α C-F bond (barrier: 4.56 kcal/mol).	The calculated adsorption energy of molecular oxygen on the defective (101) surface with OVs reaches −0.51 eV, while the value decreases to −0.35 eV on the perfect (101) surface free of OVs. Relevant bandgap parameters are also summarized for comparison.
[68]	OVs	Samples were synthesized hydrothermally and via precipitation. Characterizations include photocatalytic testing, ESR, PAL, in situ FTIR, and isotope labeling. DFT calculations used CASTEP with the GGA-PBE functional and a 380 eV cutoff.	Under simulated solar illumination, BiOBr shows optimal CO and CH_4_ production rates of 21.6 and 1.2 μmol·g^−1^·h^−1^, respectively, with nearly 100% CO selectivity at high light intensity and an apparent quantum efficiency of about 1.26%.	Adsorbed CO_2_ fills OVs, displacing neighboring Bi-O bonds. Two h^+^ then oxidize adjacent lattice oxygen to regenerate vacancies. The photoreduction proceeds via CO_2_ to COOH and then to CO + H_2_O. High-intensity irradiation suppresses CH_4_, giving nearly 100% CO selectivity.	All BiOX materials feature indirect bandgaps, and their bandgap values gradually decline with increasing atomic number of halogen X. OVs introduce intermediate bands inside the forbidden gap and flatten the VBM, accompanied by increased density of states near the band edge.
[67]	OVs	Catalysts were hydrothermally synthesized and characterized by photocatalytic tests, ESR, UV-DRS, EIS, and PL. DFT calculations gave band structure and density of states.	Photocatalytic degradation of MB and rhodamine B follows the activity sequence: BiO_0.51_F_1.98_ > BiO_0.67_F_1.66_ > Bi_7_O_5_F_11_ > BiOF. Catalytic performance rises gradually as the O/F molar ratio decreases.	Lower O/F ratio reduces defects but enhances activity: F electronegativity strengthens built-in field, suppresses recombination, and shifts bands downward (3.35–3.44 eV). EIS confirms BiO_0.51_F_1.98_ has the lowest interfacial resistance.	DFT calculations were implemented to compare the electronic structures of Bi_2_O_3_, BiOF and BiF_3_, whose calculated bandgaps are 2.24 eV, 3.40 eV and 3.70 eV respectively.
[66]	OVs	DFT calculations were conducted using the GGA–PBE functional combined with ultrasoft pseudopotentials.	Bandgap narrowing lowers excitation energy, enabling redshifted absorption of longer-wavelength photons.	OVs induce trap levels that separate electron–hole pairs, suppress recombination, and enhance photocatalytic efficiency.	DFT calculations were performed to determine the OV formation energy, analyze the electron density distribution, and compute the density of states.
[63]	Bi, O and F vacancies	DFT calculations were performed using VASP with GGA-PBE and PAW, adopting 550 eV cutoff, 3 × 3 × 4 k-point mesh, and a 2 × 2 × 2 supercell for defect modeling.	For lithium–ion battery cathode materials, DFT prediction reveals that Bi–deficient Bi_1−x_OF (x = 1/16 with Bi^3−^ vacancy) delivers the highest average intercalation voltage and theoretical specific capacity.	Pristine BiOF has a 3.10 eV bandgap and poor conductivity. Bi^3−^ vacancies narrow it to 1.56 eV, introduce mid-gap levels to aid excitation, and lower the reaction barrier to accelerate electron transfer from lithium.	The pristine BiOF exhibits a bandgap of 3.10 eV. After introducing different vacancies, the bandgap decreases to 1.56 eV for $V_{Bi}^{3-}$, 2.62 eV for $V_{O}^{2+}$, and 2.92 eV for $V_{F}^{+}$, respectively.

**Table 5:** Summary of experimental and DFT findings on intrinsic defects in BiOF.

Defect Type	DFT-Predicted Property	Experimental/Referenced Value	Agreement/Discrepancy & Critical Notes	Ref.
O vacancy	Formation energy: −4.18 eV (GGA-PBE, 2 × 2 × 1 supercell)	EPR g ≈ 2.001 signal confirms OV existence; XPS estimates ~17–18% OV content	Formation energy appears anomalously low (implies spontaneous formation), conflicting with finite experimental OV concentration; supercell convergence test and SOC correction needed.	DFT [66]; Exp [62]
O vacancy	Bandgap: 2.65 eV (vs. 3.12 eV for perfect BiOF)	Optical bandgap: 3.64–3.93 eV	GGA-PBE underestimates bandgap; hybrid functional with SOC is required for accurate defect level position.	DFT [66]; Exp [32]
O vacancy	O_2_ adsorption on (101): −0.51 eV	Enhanced RhB and PFOA photodegradation; rate constant 0.098 min^−1^ for RhB	DFT rationalizes experimental activity enhancement; transition state energy barrier (4.56 kcal/mol) supports electron-driven α-C-F cleavage.	DFT & Exp [42]
O vacancy	OV concentration correlates with (101) facet exposure	EPR intensity: BOF-3 (163 mT) > BOF-2 (55 mT) > BOF-1 (43 mT); (101)/(001) ratio: 14.87 (BOF-C)	Qualitative agreement; direct DFT comparison of OV formation energies on (101) vs. (001) facets not yet reported.	Exp [41,62]
O vacancy	Bandgap of non-stoichiometric phases: Bi_2_O_3_ (2.24 eV) < BiOF (3.40 eV) < BiF_3_ (3.70 eV)	Degradation activity: BiO_0.51_F_1.98_ > BiO_0.67_F_1.66_ > BiO_0.51_F_1.98_ [67] > Bi_7_O_5_F_11_ (MB/RhB)	OV-independent activity observed; DFT only modeled end-members, not the actual non-stoichiometric phases.	DFT & Exp
Bi vacancy	Bandgap: 1.56 eV; Average voltage: 2.25 V; Capacity: 347.6 mAh/g	No experimental report	GGA-PBE without SOC may overestimate bandgap narrowing; deep defect level may act as a recombination center; requires HSE06 + SOC verification.	DFT [63]
F vacancy	Bandgap: 2.92 eV; Formation energy higher than OVs and Bi vacancies	No experimental detection (no EPR/XPS signature reported)	Claimed impairment of internal electric field lacks direct electrostatic potential calculation; potential utility in F^−^ conduction or ESIX unexplored.	DFT [63]

**Table 6:** Summary of DFT predictions and experimental findings for various doping and substitution systems in BiOF-based photocatalysts.

Doping System	DFT Predictions	Experimental Findings	Agreement/Open Issues
In^3+^→Bi^3+^ [44]	GGA-PBE: bandgap narrowed from 3.1 to 2.7 eV; no mid-gap states	None (purely theoretical)	Lacks experimental validation; SOC omitted, Bi 5*d* not treated as valence; defect level positions remain uncertain
Sm^3+^ doping [70]	GGA-PBE: bandgap 3.197→3.045 eV; direct→indirect transition	11 mol% sample: rate constant 0.0213 min^−1^, 2.7× pristine BiOF	Trend consistent; DFT + *U* not used for 4*f* states; impurity level positions quantitatively unreliable
Eu^3+^ doping [71]	GGA-PBE + *U*: 4*f* impurity band; ^5^D_0_ level 0.28 eV below CBM	Orange-red luminescence+ enhanced photocatalysis	DFT + *U* employed for Eu 4*f*, but SOC omitted; experimental luminescence not quantitatively correlated with calculated energy gaps; level alignment requires SOC-included hybrid-functional verification
Er^3+^/Yb^3+^ co-doping [72]	GGA-PBE: direct→indirect gap; 4*f* trap states	2 mol% Yb^3+^: 95.2% (vis)/19.9% (NIR) RhB removal	UC mechanism experimentally confirmed; DFT provides static levels only, no energy transfer dynamics
Ga^3+^/Al^3+^/In^3+^/Yb^3+^ [73]	GGA-PBE: bandgap nearly unchanged (2.86–3.37 eV); diffusion barrier 0.60→0.29–0.43 eV	RT ionic conductivity 1.57 × 10^−2^–1.8 × 10^−2^ S/cm	Barrier reduction and conductivity enhancement qualitatively consistent; temperature effects not included in DFT
Bi^3+^/Te^4+^ stoichiometry [74]	GGA-PBE: BiTe_3_O_7_F direct gap ~2.92 eV	Te-rich (x = 0.12): 96% RhB/150 min	Trend qualitatively consistent; PBE absolute gap values unreliable
F^−^→Br^−^ substitution [69]	GGA-PBE: CBM shifts downward from −0.62 to −0.89 eV	BF7: 96.1% p-NP/60 min, 15× BiOBr	SOC omitted; CBM absolute values may shift by 0.3–0.5 eV with hybrid functionals; H_2_O_2_→·OH pathway lacks direct computational verification
F^−^→I^−^ substitution [43]	GGA-PBE: gap 1.78 eV, CBM = −0.75 eV	CO yield 199.2 μmol·g^−1^·h^−1^,	PBE severely underestimates the gap; CBM position is unreliable; S-scheme mechanism requires more accurate electrostatic potential calculations
O/F stoichiometry [67]	GGA-PBE on Bi_2_O_3_/BiOF/BiF_3_ end-members: gaps 2.24/3.40/3.70 eV; non-stoichiometric phases not directly modeled	BiO_0.51_F_1.98_ shows highest activity; experimental gaps: 3.35→3.44 eV as O/F ratio decreases	Non-stoichiometric phases not modeled; “enhanced built-in field” lacks direct electrostatic potential verification; experimental gap trend qualitatively matches end-member trend, but quantitative comparison is insufficient

**Table 7:** Some of the main papers discussing BiOF doping.

Ref.	Dopant Element	Doping Type	Main Mechanism	Electronic Structure Influence	Main Manifestations
[44]	In	Substitution doping	In^3+^ incorporation lowers the CBM of BiOF and narrows the bandgap without introducing mid-gap states, thereby preventing carrier trapping at defect centers.	Pristine BiOF has a ~3.1 eV direct bandgap. In-doping keeps VBM nearly constant, shifts CBM downward via Bi 6*p*/In 5*p* hybridization without in–gap states, ensuring high carrier mobility.	In-doped BiOF exhibits enhanced light absorption spanning the UV-visible region, with particularly prominent improvement in visible-light responsiveness, consequently boosting its photocatalytic performance.
[74]	Bi^3+^/Te^4+^	off–stoichiometry	Excess Te^4+^ widens the bandgap (blueshift), while surplus Bi^3+^ narrows it (redshift). Heterovalent substitution also induces lattice distortion and intrinsic defects.	The indirect–bandgap material: excess Te^4+^ induces contraction and band broadening, while surplus Bi^3+^ causes expansion and polarization, enabling bandgap tuning from 2.76 to 3.03 eV.	The Te-excess sample with (x = 0.12) achieves 96% RhB photodegradation within 150 min, and its optical absorption edge can be flexibly modulated.
[69]	F^−^	Replace with doping	F^−^ substitution introduces abundant OVs and Bi^3+^/Bi^(3−x)+^ redox couples. The upward shift in CB strengthens the reduction capability of photogenerated electrons to activate H_2_O_2_ for ·OH production, and the generated OVs further decompose H_2_O_2_ directly into ·OH.	The flattened band structure accompanies an upward shift in the CB from −0.62 eV to −0.89 eV, which strengthens the reducing capacity of photogenerated electrons; meanwhile, OVs and mixed Bi valence states facilitate efficient carrier separation.	The BiOF_0.7_Br_0.3_ sample removes 96.1% of p-nitrophenol within 60 min with an apparent reaction rate 15 times that of pristine BiOBr. Trapping experiments verify that·OH serve as the dominant reactive species.
[70]	Sm^3+^	Substitution doping	Incorporated Sm^3+^ introduces 4*f* impurity levels acting as electron trapping sites, where the generated Sm^2+^ further reduces O_2_ to yield ·O_2_^−^. Such modification extends the optical absorption range and suppresses electron–hole recombination.	The bandgap changes from direct to indirect, with Sm 4*f* impurity states appearing between VBM and CBM. Photoinduced electrons are trapped to form Sm^2+^, which then reduces O_2_ to ·O_2_^−^.	The sample doped with 11 mol% Sm^3+^ realizes complete RhB degradation within 360 min and displays favorable orange-red luminescence simultaneously.
[72]	Er^3+^, Yb^3+^	Co-doping	Yb^3+^ absorbs 980 nm NIR light and transfers energy to Er^3+^, generating visible/UV upconverted emissions reabsorbed by BiOF. Meanwhile, the 4*f* impurity levels accelerate photogenerated electron–hole separation.	After co-doping, BiOF switches from a direct- to indirect-bandgap semiconductor, with its bandgap narrowed from 3.197 eV to approximately 3.10 eV. Er/Yb 4*f*-derived impurity states emerge inside the forbidden gap and function as electron–trapping centers to restrain charge recombination.	A maximum RhB degradation efficiency of 95.2% is achieved under visible light, and prominent catalytic activity is also observed under NIR irradiation, outperforming pristine BiOF and commercial ZnO.
[71]	Eu^3+^	Doping with rare earth ions	Eu^3+^ 4*f* levels and BiOF bands determine energy transfer efficiency. Favorable alignment enables charge migration, enhancing photoluminescence and modulating photocatalytic performance.	Eu-4*f* impurity bands are embedded within the forbidden gap of BiOF. Specifically, the ^5^D_0_ excited level of Eu^3+^ lies approximately 0.28 eV below the CBM of BiOF, realizing favorable energy alignment.	Originating from the ^5^D_0_ to ^7^F_2_ transition of Eu^3+^, Eu-doped BiOF exhibits intense orange-red emission together with boosted visible–light–driven photocatalytic activity.
[73]	Ga^3+^ Al^3+^ In^3+^ Yb^3+^	Equivalent cation doping	Dopant elements induce local structural distortion, breaking the regular Bi–F tetragonal pyramid coordination in BiOF, weakening the confinement of F^−^ ions by Bi-Bi layers, and reducing the diffusion barrier of fluoride ions.	Doping leaves the bandgap of BiOF nearly unchanged (2.86–3.37 eV) and preserves its insulating nature; geometric distortion, rather than electronic modulation, is the dominant effect.	The doped Bi_0.875_M_0.125_OF (M = Ga, Al, In, Yb) exhibits a high room-temperature ionic conductivity of 1.57 × 10^−2^–1.8 × 10^−2^ S/cm with a reduced diffusion barrier of 0.29–0.43 eV.

**Table 8:** Summary of DFT predictions and experimental findings for heterojunction systems based on BiOF and related bismuth oxyfluorides.

Heterojunction System/Type	DFT Predictions	Experimental Findings	Agreement/ Open Issues
BiOBr/BiOF_0.7_I_0.3_(S-scheme) [43]	GGA-PBE: work functions BiOBr 5.06 eV, BiOF_0.7_I_0.3_ 4.73 eV; predicts electron transfer BiOF_0.7_I_0.3_→BiOBr, IEF direction BiOF_0.7_I_0.3_ → BiOBr; OVs enhance CO_2_ adsorption	In situ XPS + KPFM confirm S-scheme path; CO 199.2μmol·g^−1^·h^−1^ (10× BiOBr), CH_4_ 8.3μmol·g^−1^·h^−1^ (6.7× BiOBr)	Charge-transfer direction confirmed; GGA-PBE lacks SOC, work function accuracy uncertain; OV vs. I^−^/I^0^ contributions not distinguished
Bi_7_O_5_F_11_/BiOF(S-scheme) [78]	GGA-PBE: CBM/VBM: Bi_7_O_5_F_11_ (−1.15/2.38 eV), BiOF (−0.47/2.83 eV); electron transfer Bi_7_O_5_F_11_→BiOF; PFOA adsorption −1.33 eV (vs. −0.57 eV on BiOF)	In situ XPS + KPFM jointly verify S-scheme; PFOA (15 mg/L) completely degraded in 1 h; TOC removal 53%	Dual experimental verification; GGA-PBE lacks SOC, band-edge positions quantitatively uncertain
Bi/BiOF/Bi_2_O_2_CO_3_ (Z-scheme) [80]	GGA-PBE: bandgaps BiOF 3.14 eV, BOC 2.28 eV; work functions BiOF 5.11 eV, BOC 5.40 eV; predicts electron transfer BiOF→BOC	XPS Bi 4*f* shifts confirm electron transfer; CIP 99.8%/60 min, k = 0.0649 min^−1^ (7× BiOF, 3.5× BOC); h^+^ and ·O_2_^−^ dominant	Trend consistent; DFT only confirms static charge transfer; SPR effect not quantitatively modeled; Z-scheme assignment relies on band-edge accuracy (GGA underestimates gaps)
ZnO/BTOFYE (Z-scheme) [81]	GGA-PBE: work functions BTOFYE 4.99 eV, ZnO 5.24 eV; bandgaps BTOFYE 2.86 eV, ZnO 3.10 eV; predicts electron transfer BTOFYE→ZnO	In situ XPS + KPFM confirm Z-scheme; CIP 64%/120 min, k = 0.0085 min^−1^ (2.3× BTOFYE, 4.0× ZnO); fluorescence lifetime 85→123 μs	Z-scheme path confirmed; GGA-PBE lacks SOC; up-conversion dynamics not simulated by DFT
BiOF/BiOCl(Type-II, theoretical) [82]	PBEsol + HSE06: formation energy 50.76 meV·Å^−2^; Type-II band alignment; IEF direction BiOCl→BiOF	None (pure DFT)	IEF direction conflicts with Type-II principle; formation energy too high for feasible synthesis; no experimental validation
BiOF/BiOI(Type-II, theoretical) [82]	PBEsol + HSE06: formation energy 121.35 meV·Å^−2^; Type-II band alignment; IEF direction BiOI→BiOF	None (pure DFT)	Highest formation energy among six heterostructures; IEF direction unfavorable; no experimental validation
BiOI/BiOF(Type-I) [83]	HSE06 + vdW: formation energy +0.44 eV; fully nested band alignment; carriers accumulate on BiOI and recombine rapidly	None (pure DFT)	Type-I configuration detrimental to photocatalysis; positive formation energy indicates difficult synthesis; no experimental validation
BiOF/BiOBr (Type-I) [82]	PBEsol + HSE06: Type-I band alignment; both CBM and VBM of BiOBr nested within BiOF gap; carriers converge on BiOBr	None (pure DFT)	Theory-only prediction; subsequent I-doping of BiOF [45] circumvented this limitation, demonstrating DFT can guide doping strategies

**Table 9:** Some of the main papers discussing BiOF heterojunctions.

Ref.	Heterojunction Type	Charge Transfer Mechanism	Modification Strategy	Best Performance
[43]	S	Driven by the interfacial built-in field, electrons from BiOBr CBM recombine with holes from BiOF_0.7_I_0.3_ VBM, preserving reductive electrons in BiOF_0.7_I_0.3_ CBM and oxidative holes in BiOBr VBM.	The I^−^/I^0^ redox couple functions as a dynamic electron mediator; dual OVs boost CO_2_ adsorption and activation, and iodine doping modulates electronic band structure.	The CO and CH_4_ evolution rates reach 199.2 μmol·g^−1^·h^−1^ and 8.3 μmol·g^−1^·h^−1^, which are 10.0-fold and 6.7-fold higher than those of pristine BiOBr, respectively.
[80]	Z	Intimate contact drives electron migration from BiOF to Bi_2_O_2_CO_3_, forming an interfacial built-in field. Under light, Bi_2_O_2_CO_3_ CBM electrons recombine with BiOF VBM holes, preserving strong redox carriers.	The composite is synthesized via a one-pot route. The SPR of metallic Bi extends the light-response range, while DMF serves as both a carbon source and reducing agent.	A degradation efficiency of 99.8% is achieved within 60 min with an apparent rate constant of 0.0649 min^−1^, which is 7-fold and 3.5-fold higher than those of pristine BiOF and Bi_2_O_2_CO_3_, respectively.
[81]	Z	Work function mismatch creates an IEF from BTOFYE to ZnO. Under light, ZnO CBM electrons recombine with BTOFYE VBM holes, preserving highly redox–active carriers.	The Z-scheme heterojunction strengthens the interfacial built-in field, enhances UC luminescence, and boosts charge separation; the composite is made via electrostatic self-assembly.	A degradation efficiency of 64% is attained after 120 min, which is 2.3 times higher than pristine BTOFYE and 4.0 times higher than bare ZnO.
[83]	Type–I	Both CBM and VBM of BiOI are embedded within the bandgap of BiOF. Upon photoexcitation, photogenerated electrons and holes transfer synchronously from BiOF to BiOI and accumulate on BiOI, triggering rapid carrier recombination on BiOF and severely restraining spatial charge separation.	Face-to-face stacked vdW heterojunctions are constructed, and their band alignment is rationally modulated by the electronegativity difference between halogen atoms, where F is more electronegative than I.	Theoretical predictions reveal that BiOI/BiOF possesses a bandgap of 2.66 eV, whereas its Type-I band alignment impedes efficient separation of photogenerated electrons and holes originating from BiOF, thus restricting its practical photocatalytic utilization.
[82]	Type–II Type–I	Interfacial charge redistribution generates a built-in electric field. Electrons are lost on the BiOY side and captured on the BiOX side, yielding an electric field directed from BiOY to BiOX.	Heterojunctions are fabricated by virtue of differences in atomic radius and electronegativity of halogen atoms to tailor band alignment and interfacial built-in electric field.	Calculations show BiOCl/BiOBr has the lowest formation energy and is easy to synthesize, while BiOF/BiOY has high formation energy and poor thermodynamic stability.
[78]	S	Work function mismatch creates a built-in field; under light, BiOF CBM electrons recombine with Bi_7_O_5_F_11_ VBM holes, leaving reductive electrons on Bi_7_O_5_F_11_ CBM and oxidative holes on BiOF VBM.	BOF-2, synthesized by tuning the water/ethylene glycol ratio, is optimal; its S-scheme heterojunction provides strong surface adsorption.	PFOA (initial concentration: 15 mg/L) is completely degraded within 1 h with a TOC removal efficiency of 53%, and the catalyst exhibits stable performance over three recycling runs.

**Table 10:** Summary of DFT predictions and experimental validations for BiOF-based functional materials across diverse application fields.

Application Area	Material System	DFT-Predicted Key Parameters	Experimental Performance	Agreement/Discrepancy	Structure–Property Relationship
Photocatalytic dye degradation (RhB, MB)	(101)-facet BiOF nanoplates with OVs [41]	Bandgap 3.12→2.65 eV; OV-induced mid-gap states	Rate constant 0.098 min^−1^, 7.9× bulk BiOF; h^+^ and ·O_2_^−^ dominant	DFT explains enhanced carrier separation; OV level position not quantitatively confirmed	Surface OV density on (101) facet determines reactive species pathway and activity
	(002)-facet BiOF nanosheets [58]	IEF along c-axis; direct bandgap 3.13 eV (PBE)	Rate constant 0.025 min^−1^; h^+^ sole dominant species	IEF mechanism validated by EIS; bandgap underestimated by PBE	IEF direction normal to (002) facet enables spatial charge separation
	Sm^3+^-doped BiOF [70]	Bandgap 3.197→3.045 eV; Sm 4*f* impurity levels; direct→indirect gap	Rate constant 0.0213 min^−1^, 2.7× pristine; orange-red luminescence	Trend consistent; DFT + *U* not used for 4*f* states, level positions uncertain	4*f* impurity states introduce electron trapping and ·O_2_^−^ generation pathway
	Er^3+^/Yb^3+^ co-doped BiOF [72]	Direct→indirect gap; 4*f* trap states; narrowed bandgap	95.2% RhB removal (vis); 19.9% (NIR); up-conversion emission	UC mechanism confirmed; DFT provides static level positions only	Yb^3+^ NIR absorption + Er^3+^ up-conversion extends light harvesting to the NIR region
	Te-rich BiTe_3_O_7_F [74]	Direct gap ~2.92 eV; VBM: Bi 6*p*/O 2*p*/F 2*p*; CBM: Bi 6p/Te 5*s*	96% RhB removal in 150 min via ·O_2_^−^	PBE absolute gap unreliable; stoichiometry-activity trend consistent	Off-stoichiometry modulates lattice parameters and bandgap continuously
Photocatalytic phenolic degradation (pNP)	F-substituted BiOF_x_Br_1−x_ solid solutions [69]	CBM shifts −0.62→−0.89 eV; strengthened reduction potential	96.1% p-NP in 60 min, 15× BiOBr; ·OH dominant	CBM shift computed, but H_2_O_2_→·OH barrier not calculated	CBM edge position determines H_2_O_2_ activation and ·OH generation thermodynamics
Photocatalytic antibiotic degradation	Bi/BiOF/Bi_2_O_2_CO_3_ Z-scheme [80]	Bandgaps: BiOF 3.14 eV, BOC 2.28 eV; work functions: BiOF 5.11, BOC 5.40 eV; electron transfer BiOF→BOC	99.8% CIP in 60 min, k = 0.0649 min^−1^ (7× BiOF); h^+^ and ·O_2_^−^ dominant	DFT rationalizes static charge transfer; SPR effect not modeled	Work function difference drives IEF direction, determining Z-scheme pathway
	ZnO/Bi_3_Ti_2_O_8_F:Yb^3+^,Er^3+^ Z-scheme [81]	Work functions: BTOFYE 4.99 eV, ZnO 5.24 eV; electron transfer BTOFYE→ZnO	64% CIP in 120 min, k = 0.0085 min^−1^ (2.3× BTOFYE, 4.0× ZnO)	Z-scheme confirmed by in situ XPS/KPFM; GGA-PBE lacks SOC	IEF from work function mismatch enhances up-conversion and charge separation
Persistent pollutant degradation (PFOA/PFAS)	OV-modified (101) BiOF nanosheets [42]	O_2_ adsorption −0.35→−0.51 eV; e^−^ barrier 4.56 kcal·mol^−1^ (vs. 18.98/24.78)	Complete PFOA in 6 h, TOC 56.8%; e^−^-driven α-C-F cleavage	Barrier sequence validates e^−^-preferred pathway; PBE without SOC may affect absolute barriers	OVs enhance O_2_ adsorption; electron-initiated pathway thermodynamically most favorable
	Bi_7_O_5_F_11_/BiOF S-scheme [78]	Adsorption energy −1.33 eV vs. −0.57 eV (BiOF); CBM/VBM: −1.15/2.38 (Bi_7_O_5_F_11_), −0.47/2.83 (BiOF)	Complete PFOA in 1 h, TOC 53%; in situ XPS/KPFM verifies S-scheme	Enhanced adsorption confirmed; band edge positions from GGA-PBE without SOC	Strong adsorption + S-scheme IEF enables complete mineralization with high efficiency
	In-MOF/BiOF heterojunction [84]	Adsorption energy order: PFOS > 6:2 Cl-PFESA > PFOA > HFPO-TA; electron transfer BiOF→In-MOF	PFOS 100% in 90 min; PFOA 100% in 150 min; h^+^ and ·O_2_^−^ dominant	Rate constants correlate with adsorption energy; PFAS degradation pathways not computed	In-MOF provides adsorption sites; IEF at interface drives carrier separation
CO_2_ photoreduction	BiOBr/BiOF_0.7_I_0.3_ S-scheme [43]	Work functions: BiOBr 5.06 eV, BiOF_0.7_I_0.3_ 4.73 eV; electron transfer BiOF_0.7_I_0.3_→BiOBr	CO 199.2 μmol·g^−1^·h^−1^, CH_4_ 8.3 μmol·g^−1^·h^−1^ (10×/6.7× BiOBr)	S-scheme confirmed by in situ XPS/KPFM/fs-TA; GGA-PBE work function accuracy uncertain	Dual OVs + I^−^/I^0^ redox couple + S-scheme IEF create a synergistic CO_2_ reduction pathway
	BiOX series comparison (X = F, Cl, Br, I) [68]	OVs introduce mid-gap states; flatten VBM; activity: BiOBr > BiOCl > BiOI > BiOF	BiOBr highest CO yield (21.6 μmol·g^−1^·h^−1^); BiOF lowest	In situ FTIR confirms OV-mediated pathway; BiOF limited by wide gap and weak CO_2_ adsorption	OV concentration and electron population around lattice O determine CO_2_ activation efficiency
Electrochemical energy storage (Li-ion battery)	Bi-deficient Bi_1−x_OF [63]	Bandgap 3.10→1.56 eV; avg voltage 2.25 V; capacity 347.6 mAh/g	No experimental validation; purely theoretical prediction	PBE without SOC; deep defect level may act as a recombination center rather than a conductivity enhancer	Bi vacancies introduce Bi 6*p* mid-gap states that may improve conductivity, but experimental verification is needed
F^−^ adsorption/electro-sorption	BiOF with tunable (101) facet exposure [62]	Adsorption energy on (101): −3.91 eV vs. (001): −3.16 eV; OV-enhanced surface reactivity	Adsorption capacity 24.14 mg·g^−1^ at 0.9 V; selectivity F^−^/Cl^−^ 6.585	Adsorption energy difference correlates with facet-dependent selectivity	(101) facet exposure enhances F^−^ capture through stronger adsorption energetics; applied potential triggers reversible capture/release

## Data Availability

Data will be made available on request.
